# Reaching early adolescents with a complex intervention for HIV prevention: findings from a cohort study to evaluate DREAMS in two informal settlements in Nairobi, Kenya

**DOI:** 10.1186/s12889-021-11017-y

**Published:** 2021-06-10

**Authors:** Sarah Mulwa, Jane Osindo, Elvis O. Wambiya, Annabelle Gourlay, Beatrice W. Maina, Benedict O. Orindi, Sian Floyd, Abdhalah Ziraba, Isolde Birdthistle

**Affiliations:** 1 Faculty of Epidemiology and Population Health, London School of Hygiene & Tropical Medicine, London, UK; 2 African Population and Health Research Center, Nairobi, Kenya; 3 Center for Geographic Medicine Research, Kenya Medical Research Institute − Wellcome Trust Research Programme, Kilifi, Kenya

**Keywords:** Early adolescent girls, HIV prevention, Complex interventions, Implementation, Impact evaluation

## Abstract

**Background:**

The DREAMS Partnership promotes combination HIV prevention among adolescent girls and young women. We examined the extent to which DREAMS interventions reached early adolescent girls (EAG; aged 10–14 years) in two informal settlements in Nairobi, and the characteristics of those reached, after 3 years of implementation.

**Methods:**

We utilized three data rounds from a randomly-sampled cohort of EAG established in 2017 in Korogocho and Viwandani informal settlements where DREAMS interventions were implemented. Interventions were classified as individual or contextual-level, with individual interventions further categorised as primary (prioritised for this age group), or secondary. We summarised self-reported invitation to participate in DREAMS, and uptake of eight interventions that were supported by DREAMS, during 2017–2019. Multivariable logistic regression analysis was used to identify individual and household characteristics associated with invitation to DREAMS and uptake of primary interventions.

**Results:**

Data were available for 606, 516 (retention rate of 85%) and 494 (82%) EAG in 2017, 2018 and 2019, respectively. Proportions invited to DREAMS increased from 49% in 2017, to 77% by 2018, and to 88% by 2019. School-based HIV and violence prevention, and HIV testing and counselling were the most accessed interventions (both at 82%). Cumulative uptake of interventions was higher among those invited to participate in DREAMS compared to those never invited, particularly for new interventions such as social asset building and financial capability training. Contextual-level interventions were accessed infrequently. Most of those invited both in 2017 and 2018 accessed ≥3 interventions (96%), and 55% received all three primary interventions by 2019.

**Conclusions:**

Uptake of DREAMS interventions among a representative sample of EAG was high and quickly increased over the implementation period. The majority accessed multiple interventions, indicating that it is feasible to integrate and deliver a package of interventions to EAG in a challenging informal context.

**Supplementary Information:**

The online version contains supplementary material available at 10.1186/s12889-021-11017-y.

## Introduction

Adolescent girls and young women (AGYW) aged 15–24 years remain at higher risk of HIV infection compared to their male peers, especially in sub-Saharan Africa [1, 2]. Sources of HIV risk among AGYW are varied and include limited access to sexual and reproductive health services, economic pressures driving transactional sex and experiences of violence [3–7]. A proliferating, broad evidence base around these vulnerabilities has informed the recent expansion of HIV programming for AGYW [5]. In contrast, equivalent data among early adolescent girls (EAG) aged 10–14 years are sparse, in part due to low HIV incidence [2], and HIV prevention approaches for this age group are less well understood [3, 8–11]. However, early adolescence presents a key opportunity to intervene that can result in sustained health, education and social-economic benefits [12].

Adolescents living in informal settlements face distinct challenges as they transition to adulthood. Urban informal contexts are often characterized by food insecurity, violence, inadequate housing, and poor sanitation and infrastructure [3, 6, 13, 14]. In informal settlements in Nairobi for instance, six in ten girls aged 10–14 years have experienced violence [15]. Girls in these settings are also at high risk of dropping out of school [16]. While much has been done to document health outcomes in these contexts, EAG continue to be under-represented in research, perhaps due to perceived difficulties and ethical issues around recruiting and engaging this age group [8, 17]. Projects such as the Global Early Adolescent Study (GEAS) are creating new approaches and opportunities for conducting research with EAG, by contributing to appropriate data collection tools and methods [17, 18]. Formative studies from GEAS, including research in informal settlements, show that gender socialization occurs early in life, and that interventions encouraging the development of positive attitudes and equitable gender norms can be transformative immediately and over the life course [18].

Intervening early can also ensure that EAG are reached with prevention messages and skills that promote healthy sexual relationships and behaviours before they transition to higher risk groups, when incidence of HIV, STI and pregnancy escalate [5, 19]. Developing holistic interventions that promote learning, self-efficacy, life-skills, and positive social norms, and that involve caregivers who play a key role in shaping the attitudes of adolescents, are key to the life-long development and health trajectory of EAG [20].

Agencies are increasingly investing in early adolescence, with a focus on delivery of multi-sectoral and comprehensive programmes [16, 21–23]. As a response to repeated calls to invest in combination interventions and accelerate HIV prevention programming, the DREAMS (Determined, Resilient, Empowered, AIDS-free, Mentored and Safe girls) Partnership promotes a ‘layered’ package of evidence-based, multi-sectoral interventions to AGYW aged 10–24 years, with a primary aim of reducing HIV acquisition [5]. DREAMS was implemented in countries with high HIV burden, and targeted vulnerable AGYW in the most affected sub-national units (district-level) including urban informal settlements. DREAMS is based on the principle that HIV prevention will be most effective when it targets the myriad of behavioural, social and structural factors driving HIV risk [5]. While the value of intervening early in adolescence is recognised, there is limited documentation of whether and how complex interventions can be delivered and scaled up to the intended participants [24, 25]. A recent journal supplement on ‘data driven HIV prevention’ calls for researchers and program implementers to “learn how to best deliver combination and multi-dimensional programmes for youth” [25].

We sought to explore the awareness, reach and uptake of DREAMS interventions, and the profile of EAG reached by the programme after 3 years of implementation, in two urban informal settlements in Kenya.

## Methods

### Panel box: implementation of DREAMS interventions

DREAMS interventions were delivered by implementing partners (IPs) contracted by United States (US) Government agencies [26]. One IP coordinated the delivery of all interventions in each informal settlement, where most DREAMS interventions were introduced in early 2016 (Pre-Exposure Prophylaxis was introduced in 2017) [26]. Implementation was staggered, with interventions which had pre-existing infrastructure the first to roll out e.g., HIV testing services. Newer services e.g., social asset building, took slightly longer to introduce as IPs needed time for training and adapting the interventions to the local context.

IPs sought to reach and invite the most vulnerable EAG to participate in DREAMS e.g., by inviting those who were food insecure, of school-age and out of school, orphaned or those who had ever been pregnant. Vulnerable EAG were identified through the ‘Girl Roster’ census method [27]. The method involved collection of data on households, and status of girls in those households in a given community, which was operationalised through door-to-door home visits. The girls were classified into segments based on various characteristics (e.g., orphan, or in/out of school). The Girl Roster method identified more potential beneficiaries than resources would allow, and was supplemented by other approaches to identify those in the highest-risk group (e.g., through consultation with community members) [26]. DREAMS IPs extended invitation to participate in DREAMS through 2018, with continued programming among those who had been invited and agreed to participate. No compensation was offered to participate in the interventions.

Table 1 summarises the interventions available to EAG aged 10–14 years, classified broadly into two levels (individual or contextual-level interventions). Individual-level interventions were further grouped into primary or secondary interventions as per the US President’s Emergency Fund for AIDS Relief (PEPFAR) guidance to countries [28]. ‘Primary’ interventions are those considered by PEPFAR as a priority for EAG, while ‘secondary’ interventions are based on individual need or circumstance, e.g., EAG who experienced violence should receive post-violence care services.

**Table 1:** Summary of DREAMS funded interventions among 10–14 year olds in Nairobi, grouped as either individual-level (primary or secondary) or contextual-level interventions

(A) Level	(B) Sub-category	(C) Intervention	(D) Description of activities & interventions relating to DREAMS implementation	(E) Specific activities and interventions as measured through DREAMS Impact Evaluation surveys in Nairobi
**Individual**	**Primary individual interventions**	Social asset building^a^	Build social skills and networks; connect AGYW with peers, mentors & adults for information, emotional & material support	Awareness of and participation in a safe space program
		School-based HIV and violence prevention^b^	HIV & sex education, violence prevention education in schools	Awareness of and participation in school based HIV educational programs and violence prevention programs
		Financial capability training^b^	Financial capability training	Awareness of and participation in financial literacy training for girls or young women
	**Secondary individual interventions**	HIV testing and counselling services ^a^	HIV testing; partner testing if necessary; linkage to care & ART if positive, or linkage to other DREAMS prevention interventions if negative	Awareness of and participation in HIV testing, partner testing, or linkage to ART
		Post-violence care services^a^	Youth-friendly screening & care for intimate partner violence/ violence against children, provision of Post Exposure Propylaxis (PEP)	Awareness of and participation in post-violence care services, HIV/STI testing after violence or PEP
		Education subsidies^a^	Support with school fees, uniforms etc. to enhance secondary school enrolment and retention for vulnerable girls	Awareness of and participation in educational subsidies (e.g support for school fees, uniforms, books or stationery) to help girls stay in school.
**Range of individual level interventions**		**3–6**		
**Contextual**	**Contextual level interventions**	Parenting/caregiver programmes^b^	Parenting programmes on adolescent sexual/risk behaviours & protection from violence	Awareness of and participation in parenting/caregiving programs like Families Matter Program
		Community mobilisation and norms change^a^	Community-based HIV and violence prevention programmes, social/gender norms change & gender-related messaging	Awareness of and participation in violence prevention and gender norms –related training or education in the community
**Total contextual level interventions**		**2**		
**Total DREAMS funded interventions for 10–14 year olds: 8**				

^a^These interventions were offered on a continous basis - social asset building meetings occurred weekly, and the EAG were encouraged to attend as many meetings as possible, for HIV services girls were retested every 6 months after the first test, post violence care services were provided if there was a report of violence, education subsidies were provided for as long as the beneficiary was still in school and qualified to receive it, while community mobilization involved engaging community members through meetings or discussion forums; ^b^ These interventions were session based - school-based HIV and violence prevention, and financial capability training were planned for 7 sessions, while parenting programs were planned for 5 sessions

Individual-level interventions were delivered directly to EAG and aim to empower girls, for example, through social asset building interventions where EAG build networks with peers and receive information and support from mentors. These interventions were typically offered to groups of girls in safe spaces (defined as a girl-only space, which could be in church halls or community centres), or in other venues (e.g., schools after regular school hours). Provision of HIV testing, or information on condoms were typically offered in safe spaces or the field offices of IPs, while referrals for clinical services were made to primary health care facilities [26]. Contextual-level interventions were intended to strengthen families (e.g., through parenting programs to enhance positive relationships with their children), and to mobilise communities to address social norms for violence and HIV prevention.

Up to eight interventions were available for EAG invited to DREAMS (Table 1). Three interventions were curriculum-based: school-based HIV/violence prevention and financial capability training were each planned for 7 sessions; parenting programs for 5 sessions. The other interventions were to be offered continuously as appropriate, e.g., DREAMS invitees were encouraged to attend social asset building meetings as long they continued to engage in the program (Table 1). When EAG transitioned to age 15, they became eligible for the DREAMS interventions offered to older AGYW aged 15+ years (e.g., contraception counselling).

### Evaluation study design, setting and sample

To assess the reach and uptake of DREAMS interventions, and the profile of DREAMS participants, we conducted three annual rounds of interviews with a representative cohort of EAG aged 10–14 years in Korogocho and Viwandani informal settlements of Nairobi. This cohort was part of a larger, independent study to evaluate the impact of DREAMS interventions in Nairobi and three other settings (in Kenya, South Africa and Zimbabwe) [4]. In brief, the cohort participants were randomly selected from the Nairobi Urban and Health Demographic Surveillance System (NUHDSS) sampling framework, a general population research platform which profiles residents of the two urban informal settlements [29].

At enrolment in 2017, we targeted a minimum sample of 500 girls. A random list of potentially eligible girls was generated from the most recent NUHDSS survey, and attempts were made to reach all girls in the list. Data were collected using an electronic interviewer-administered questionnaire (Additional file 1). The questionnaire was developed by the research team, and comprised questions on gender norms, adolescent health and behaviour, schooling status, experiences of violence, and sexual debut among others. Some measures were adapted from a toolkit developed in the context of GEAS [30]. DREAMS-specific questions covered self-reported invitation to participate in DREAMS activities, awareness of, and participation in each DREAMS intervention. The tools were pre-tested during a pilot in February 2017. Data were collected between March–July 2017, July–December 2018 and May–August 2019. Interviews were conducted in a secure, private location at the field research office.

### Measures

We used self-reported invitation to participate in DREAMS in the first two rounds of interview (2017 and/or 2018) to classify respondents as DREAMS beneficiaries or not (‘Invited to DREAMS by 2018’, yes/no). We also defined cumulative exposure to DREAMS in three categories classified as never invited by 2018 (‘never invited’), newly invited in 2018, and invited both in 2017 and 2018 (i.e., those who reported being invited in 2017 only, or in 2017 and 2018). We used this variable to capture the timing of recruitment into DREAMS, in order to identify differences in those invited earliest (e.g., since 2017) compared to those invited later, since IPs may have altered their targeting criteria over time. Uptake of each intervention in each year was assessed based on the response to the question: Did you participate in intervention X in the past 12 months? (yes/no). For curriculum-based interventions such as financial capability training, uptake comprised participation in at least one session.

### Participant characteristics

Informed by the criteria used by IPs to recruit participants into DREAMS interventions, socio−demographic variables at cohort enrolment in 2017 were selected for the profiling analysis. Variables included age-group, settlement site, household food insecurity, ever had romantic relationships, experienced violence in the previous 6 months, ever sexually exploited, and paid work in the previous 6 months. Expected school grade was based on the level at which a respondent should be given her age, had she begun primary school at age six.

### Analysis

Analyses were done in Stata/SE version 15 and restricted to girls interviewed in 2019. Frequencies and percentages were used to summarize awareness of, invitation to participate in, and uptake of DREAMS interventions, within the 12 months preceding the survey as well as cumulatively. The median (interquartile range) number of sessions attended and the proportions completing all sessions for the curriculum-based interventions were also calculated.

To assess evidence of layering, we summarised: (a) proportions who received multiple primary interventions (≥2 or all 3), (b) proportions who received multiple interventions by 2019 (i.e., more than one primary, secondary and/or contextual-level intervention); and (c) combined participation in interventions from across different levels (i.e., received a combination of individual-level and contextual-level interventions).

To profile the characteristics of girls reached by DREAMS, logistic regression models were fitted. Odds ratios (OR) and 95% confidence intervals (CIs) were used to quantify the strength of association between individual/household characteristics and measures of DREAMS uptake, specifically (i) invitation to participate in DREAMS by 2018, and (ii) uptake of multiple primary interventions (0–2 vs all three) by 2019. All variables independently associated with each outcome at *p* ≤ 0.2 were included in the final multivariable model in a forward step-wise approach, starting with the variables with the strongest association with the outcome. Age and site were included a priori. The STROBE guidelines were used in synthesising and reporting these results [31].

### Ethics approval

Ethics approval was obtained from AMREF Health Africa Ethics and Scientific Review Committee (ESRC) (AMREF; No ESRC P298/2016) and the London School of Hygiene & Tropical Medicine (LSHTM; Ref 11835). An information sheet was used to inform potential participants and their parents/guardians about the study. Written informed parental/guardian consent and participant assent were obtained before commencing an interview. Since interviews were conducted in a safe site outside of respondents’ homes, compensation was provided to cover transport costs and snacks.

## Results

### Participation rates and participant characteristics

Out of the random list of 1017 EAG eligible to participate in the evaluation study at enrolment, 333 (33%) were no longer eligible at the time of visit, e.g., due to out-migrations and age ineligibility. Of the remaining 684, 46 (7%) were absent for extended periods of time, 23 (3%) had their structures located but respondents’ whereabouts were unknown, and 9 (1%) refused to participate, leaving 606 (89%) who consented and completed the interviews.

Of the 606 respondents enrolled in 2017, 516 (85%) and 494 (82%) were followed up and interviewed in 2018 and 2019 respectively. Respondents from Viwandani were more likely than those from Korogocho to be lost to follow-up, while single/double orphans were more likely to be lost to follow-up than non-orphans (Additional file 2).

Almost all respondents followed up in 2019 were attending school at the time of cohort enrolment, and the majority were aged 10–12 years (62%), resident in Korogocho (57%), and reported food insecurity (62%). About 40% had experienced any physical, verbal or sexual violence at the time of enrolment (Table 2). Patterns of invitation to participate in DREAMS over time were largely similar by baseline characteristics, although higher proportions of those aged 10–12 years, and from Viwandani were newly invited in 2018 (Table 2).

**Table 2:** Baseline cohort profile among EAG retained and interviewed in 2019 by invitation to participate in DREAMS

	Full sample		Invitation status		
	Total (N)	%	Never invited: n^**a**^ (%)	Invited both in 2017 and 2018: n^**a**^ (%)	Newly invited in 2018: n^**a**^ (%)
**Total**	494	–	114 (23.1)	243 (49.2)	137 (27.7)
**Age group**					
10–12	307	62.1	71 (23.1)	141 (45.9)	95 (30.9)
13–14	187	37.9	43 (23.0)	102 (54.5)	42 (22.5)
**Informal settlement area**					
Korogocho	280	56.7	52 (18.6)	166 (59.3)	62 (22.1)
Viwandani	214	43.3	62 (29.0)	77 (36.0)	75 (35.0)
**Currently enrolled in school**					
No	5	0.8	3 (75.0)	1 (25.0)	0 (0.0)
Yes	601	99.2	111 (22.7)	242 (49.4)	137 (28.0)
**Current schooling and school progress**					
2+ classes behind	150	30.4	31 (20.7)	85 (56.7)	34 (22.7)
< 2 classes behind	344	69.6	83 (24.1)	158 (45.9)	103 (29.9)
**Orphanhood status**					
Not an orphan	428	86.6	100 (23.4)	207 (48.4)	121 (28.3)
Single/double orphan	66	13.4	14 (21.2)	36 (54.6)	16 (24.4)
**Paid jobs/activities, last 6 months**					
No	470	95.1	106 (22.6)	231 (49.1)	133 (28.3)
Yes	24	4.9	8 (33.3)	12 (50.0)	4 (16.7)
**Family food insecurity**^b^					
Never	188	38.1	53 (28.2)	82 (43.6)	53 (28.2)
Sometimes	267	54.0	55 (20.6)	136 (50.9)	76 (28.5)
Often	39	7.9	6 (15.4)	25 (64.1)	8 (20.5)
**Romantic relationships**					
Never been in a relationship	445	90.3	100 (22.5)	223 (50.1)	122 (27.4)
Ever been in a relationship	48	9.7	14 (29.2)	19 (39.6)	15 (31.3)
**Sexually exploited**^c^					
No	463	93.7	107 (23.1)	231 (49.9)	125 (27.0)
Yes	31	6.3	7 (22.6)	12 (38.7)	12 (38.7)
**Physical violence, last 6 months**					
No	414	83.8	93 (22.5)	208 (50.2)	113 (27.3)
Yes (slapped, hit, physically hurt)	80	16.2	21 (26.3)	35 (43.8)	24 (30.0)
**Verbal violence, last 6 months**					
No	327	66.2	72 (22.0)	167 (51.1)	88 (26.9)
Yes (teased, bullied or threatened)	167	33.8	42 (25.1)	76 (45.5)	49 (29.3)

^**a**^number in the indicated invitation category; ^b^ ever been a time when your family did not have enough food because they had no money; ^c^reported being threatened, coerced or being forced into being touched or having (first) sex, or said they were unwilling to have (first) sex, or they were ever forced into/attempted sex by an adult (childhood experiences), or reported being touched in the last 6 months in a way they did not want to be touched

### Awareness of DREAMS interventions

Awareness of DREAMS was high in 2017 after approximately 1 year of DREAMS implementation, with 82% of participants reporting they had heard about DREAMS and 49% having been invited to participate in DREAMS activities (Additional file 3). In 2019, cumulative awareness and invitation to participate increased to > 99 and 88% respectively. Awareness of specific DREAMS interventions in each year was generally high, more so for individual-level interventions than contextual-level interventions.

### Uptake of DREAMS interventions

**Fig. 1:**
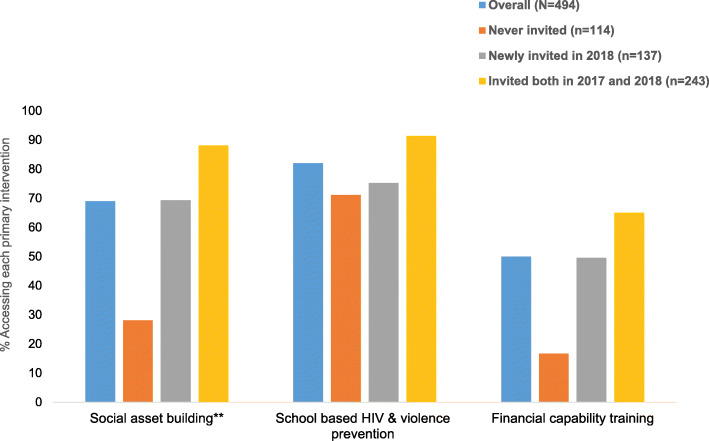
Cumulative uptake by 2019* of primary interventions among 10–14 year olds followed up in 2019 by invitation to participate in DREAMS. *Participated in the intervention either in 2017 or 2018 or 2019. **Social asset building includes interventions aimed at building social skills and networks, connecting adolescent girls and young women with peers, mentors and other adults for information, emotional and material support

The most accessed primary intervention by 2019 was school-based HIV and violence prevention (82%), followed by social asset building (69%) and financial capability training (50%). Cumulative uptake of primary interventions was higher among those invited to participate in DREAMS compared to those never invited. There was an increasing trend in uptake by length of participation in DREAMS, especially for social asset building and financial capability training. Uptake of school-based HIV and violence prevention was also high among those never invited into DREAMS (71%) (Fig. 1).

**Fig. 2:**
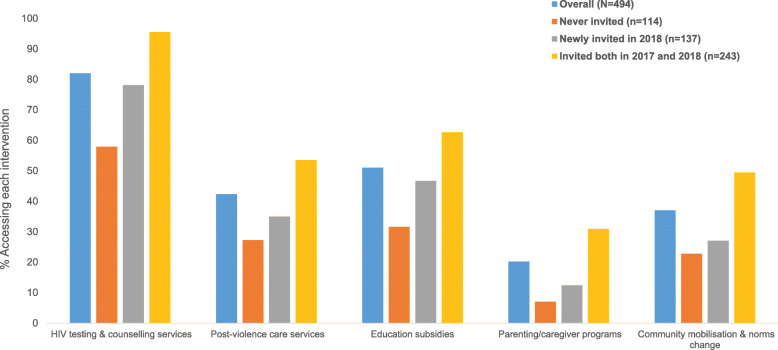
Cumulative uptake by 2019* of secondary and contextual level interventions among 10–14 year olds by invitation to participate in DREAMS. *Participated in the intervention either in 2017 or 2018 or 2019

We also found high cumulative uptake of secondary interventions, with those invited to DREAMS reporting higher uptake compared to those never invited. For instance, cumulative uptake of HIV testing and counselling, educational subsidies and post-violence care services was 96, 63 and 54% respectively among those invited to DREAMS both in 2017 and 2018, compared to 58, 32 and 27% among those never invited. The most accessed secondary intervention was HIV testing and counselling (82%) (Fig. 2).

Uptake of contextual-level interventions, i.e., community mobilisation and parenting programs, was also markedly higher among those invited to DREAMS compared to those never invited (e.g., 31% uptake for parenting programs among those invited both in 2017 and 2018 compared to 7% among those never invited) (Fig. 2). For most interventions, uptake within the last 12 months increased between 2017 and 2018, but remained largely unchanged between 2018 and 2019 (Additional file 4).

In 2019, the median (interquartile range) number of sessions attended in the past 12 months were: school-based HIV and violence prevention 4 [2, 6] of a total 7; financial capability training 3 [2, 6] of total 7; and parenting programs 2 [1, 3] out of 5. The median number of sessions for social asset building, which was offered on a continuous basis and did not have a limit was 4 [2, 10]. Proportions accessing all intended sessions of a curriculum were low overall: school-based HIV and violence prevention (21%); financial capability training (14%), and parenting programs (8%).

### Uptake of multiple interventions

**Fig. 3:**
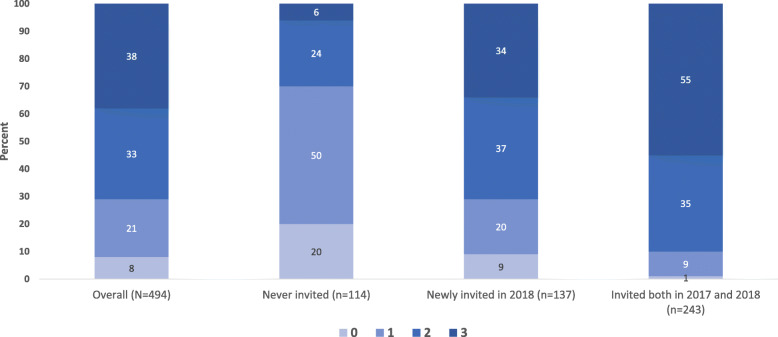
Number of primary interventions accessed by 2019*, overall and by invitation to participate in DREAMS. *Total number of primary interventions accessed by 2019. Primary interventions are social asset building, school based HIV & violence prevention, and financial capability training

Participation in multiple primary interventions increased over time and by length of invitation to DREAMS. Cumulatively by 2019, > 70% of study participants had accessed ≥2 primary interventions, while 38% had accessed all three, with significantly higher proportions among those invited to DREAMS (e.g., 55% among those invited both in 2017 and 2018, and 34% among those newly invited in 2018) compared to those never invited (6%) (Fig. 3). Among those accessing two primary interventions, social asset building and school-based HIV and violence prevention were the most common combinations (Additional file 5).

When participation in all eight interventions in Table 1 was considered, the majority of EAG (80%) had accessed at least three by 2019, with participation markedly higher among those invited to DREAMS compared to those never invited (90% among those ever invited; 46% among never invited) (Additional file 6). Combinations across the individual and contextual-levels were less frequent, with 11% of those who had accessed any individual-level intervention also accessing parenting plus community interventions. Combinations of interventions also increased by year of reported invitation to DREAMS, with 17% of those invited to DREAMS both in 2017 and 2018 accessing interventions across individual, family and community level (2% of those never invited; 6% of those newly invited in 2018) (Additional file 7).

### Uptake by characteristics of EAG

Patterns of invitation to DREAMS were broadly similar by baseline characteristics after 3 years of implementation (Table 2, Table 3). Based on univariable analyses of invitation to DREAMS by 2018, EAG were more likely to be invited if they had experienced food insecurity, and less likely to be invited if they were from Viwandani (Table 3). In multivariable analyses, there remained evidence of an association between study site with invitation to DREAMS (adjusted OR (aOR) = 0.60; 95%CI 0.39, 0.93), while the association between invitation and experiences of food insecurity weakened.

**Table 3:** Univariable and multivariable analyses of baseline characteristics associated with being invited to participate in DREAMS among EAG followed up in 2019

EAG characteristics	Total (N)	Number invited by 2018 (n)	Proportion invited by 2018 (%)	cOR	95% CI		p (LRT)	aOR	95% CI		p (LRT)
**Age group**											
10–12	307	236	76.9	1				1			
13–14	187	144	77.0	1	0.7	1.6	0.973	1	0.6	1.5	0.958
**Informal settlement area**											
Korogocho	280	228	81.4	1				1			
Viwandani	214	152	71.0	0.6	0.4	0.9	0.007	0.60	0.4	0.9	0.023
**Current schooling and school progress**											
2+ classes behind	150	119	79.3	1							
< 2 classes behind	344	261	75.9	0.8	0.5	1.3	0.402				
**Orphanhood status**											
Not an orphan	428	328	76.6	1							
Single/double orphan	66	52	78.8	1.1	0.60	2.1	0.699				
**Paid jobs/activities, last 6 months**											
No	470	364	77.4	1							
Yes	24	16	66.7	0.6	0.2	1.40	0.226				
**Family food insecurity**^a^											
Never	188	135	71.8	1				1			
Sometimes	267	212	79.4	1.5	1	2.3		1.3	0.9	2.10	
Often	39	33	84.6	2.2	0.9	5.5	0.081	1.9	0.8	4.9	0.235
**Romantic relationships**											
Never been in a relationship	445	345	77.5	1							
Ever been in a relationship	48	34	70.8	0.70	0.4	1.4	0.298				
**Sexually exploited**^b^											
No	463	356	76.9	1							
Yes	31	24	77.4	1	0.4	2.5	0.946				
**Physical violence, last 6 months**											
No	414	321	77.5	1							
Yes (being slapped, hit, physically hurt)	80	59	73.8	0.8	0.5	1.4	0.462				
**Verbal violence, last 6 months**											
No	327	255	78.0	1							
Yes (teased, bullied or threatened)	167	125	74.9	0.8	0.5	1.30	0.435				

*cOR* crude odds ratio; *aOR* adjusted odds ratio; *LRT* likelihood ratio test; ^a^ ever been a time when your family did not have enough food because they had no money; ^b^reported being threatened, coerced or being forced into being touched or having (first) sex, or said they were unwilling to have (first) sex, or they were ever forced into/attempted sex by an adult (childhood experiences), or reported being touched in the last 6 months in a way they did not want to be touched

Access to all three primary interventions did not differ by socio-demographic characteristics such as orphanhood, food insecurity or being behind at school (Table 4). However, those who had ever been in a romantic relationship (aOR = 0.46; 95%CI 0.23, 0.94) or experienced verbal violence (aOR = 0.68; 95%CI 0.45, 1.01) (weak evidence), were less likely to access all three primary interventions.

**Table 4:** Univariable and multivariable analyses of characteristics associated with participation in three primary interventions by 2019 among EAG in Nairobi

	Participation in three primary interventions by 2019^a^														
								Age and site adjusted				Fully adjusted model			
**EAG characteristics**	**Total (N)**	**Number who accessed three primary interventions (n)**	**Proportion who accessed three primary interventions (%)**	**cOR**	**95% CI**		**p (LRT)**	**aOR**	**95% CI**		**p (LRT)**	**aOR**	**95% CI**		**p (LRT)**
**Age group**															
10–12	307	109	35.5	1				1				1			
13–14	187	78	41.7	1.30	0.90	1.89	0.17	1.30	0.90	1.89	0.16	1.39	0.95	2.03	0.09
**Informal settlement area**															
Korogocho	280	110	39.3	1				1				1			
Viwandani	214	77	36.0	0.87	0.6	1.26	0.45	0.86	0.60	1.25	0.44	0.87	0.60	1.26	0.45
**Current schooling and school progress**															
2+ classes behind	150	52	34.7	1				1							
< 2 classes behind	344	135	39.2	1.22	0.82	1.82	0.34	1.32	0.86	2.03	0.2				
**Orphanhood status**															
Not an orphan	428	161	37.6	1				1							
Single/double orphan	66	26	39.4	1.08	0.63	1.83	0.78	1.06	0.62	1.80	0.84				
**Paid jobs/activities, last 6 months**															
No	470	176	37.4	1				1							
Yes	24	11	45.8	1.41	0.62	3.22	0.411	1.37	0.60	3.13	0.459				
**Family food insecurity**^b^															
Never	188	63	33.5	1				1							
Sometimes	267	106	39.7	1.31	0.88	1.93		1.26	0.84	1.88					
Often	39	18	46.2	1.70	0.85	3.42	0.220	1.64	0.81	3.32	0.313				
**Romantic relationships**															
Never been in a relationship	445	176	39.6	1				1				1			
Ever been in a relationship	48	11	22.9	0.45	0.23	0.91	0.03	0.42	0.21	0.86	0.02	0.46	0.23	0.94	0.03
**Sexually exploited**^c^															
No	463	178	38.4	1				1							
Yes	31	9	29	0.66	0.29	1.45	0.3	0.66	0.3	1.48	0.32				
**Physical violence, last 6 months**															
No	414	161	38.9	1				1							
Yes (being slapped, hit, physically hurt)	80	26	32.5	0.76	0.46	1.26	0.28	0.77	0.46	1.28	0.32				
**Verbal violence, last6 months**															
No	327	135	41.3	1				1				1			
Yes (teased, bullied or threatened)	167	52	31.1	0.64	0.43	0.95	0.03	0.64	0.43	0.95	0.03	0.68	0.45	1.01	0.06

^a^Based on the number of primary interventions that the girls self-reported participating in within the last 12 months, either in 2017, 2018 or 2019; *cOR* crude odds ratio; *aOR* adjusted odds ratio; *LRT* likelihood ratio test; ^b^ ever been a time when your family did not have enough food because they had no money; ^c^reported being threatened, coerced or being forced into being touched or having (first) sex, or said they were unwilling to have (first) sex, or they were ever forced into/attempted sex by an adult (childhood experiences), or reported being touched in the last 6 months in a way they did not want to be touched

## Discussion

We assessed the extent to which a complex, multi-sectoral intervention for HIV prevention (DREAMS) reached early adolescent girls in two urban informal settlements in Kenya, and found evidence of strong programme penetration among a representative sample of girls. Uptake of each primary intervention was high ranging from 50% (financial capability training) to 82% (school-based HIV and violence prevention) indicating that interventions for EAG can be delivered and integrated across schools and community-based settings [32–34]. Uptake was high among DREAMS invitees for interventions that were newly introduced into this setting by DREAMS, such as financial capability training and social asset building (reaching 59 and 81% of EAG invited into DREAMS respectively), as well as interventions that existed prior to, or outside of DREAMS. High uptake of the latter, such as school-based HIV and violence prevention among non-DREAMS invitees, may reflect access through other programmes and funders.

Girls’ receipt of multiple interventions improved over time and more so among DREAMS invitees compared to non-invitees, indicating that with sustained effort over multiple years, integration of a coherent package of interventions is feasible and can be scaled up to reach a majority of EAG in environments with limited resources and infrastructure. While few studies have provided information on service uptake among early adolescents, particularly for complex interventions [32, 35, 36], previous research has shown the value of sustained investment. For example, the evaluation of the Gender Equity Movement in Schools (GEMS) program among young adolescents aged 12─14 years concluded that longer periods of programming could improve the effectiveness of the program [36]. Another study, assessing large-scale replicability of a programme training teachers to deliver HIV education to upper primary school pupils in Kenya, found that both teaching practices and learning improved with time [37], suggesting that sustained commitment to programming will improve uptake as well as outcomes.

Most of the DREAMS interventions accessed were at the individual level, with combinations of individual and contextual-level interventions less common. This is because EAG participation in parenting and community mobilization interventions was relatively low (compared to individual-level interventions) and more can be done to expand access. A related study reported low uptake of DREAMS’ parenting and community programs among older AGYW aged 15–22 years, as well as older women and men in the community [38, 39]. Given the important role of parents, male partners and the broader community in shaping adolescent health and development, low uptake of contextual interventions is likely to limit the effectiveness of DREAMS, and is an important area for improvement.

For curriculum-based interventions, we found that few EAG attended the total number of sessions provided, although half of the participants accessed at least ≥3 sessions for school-based HIV and violence prevention and financial capability training. Sessions on parenting programs were attended less frequently. This shows that it may be more feasible to engage EAG and parents in shorter, intense programmes rather than longer ones. This finding is echoed in our related qualitative research with IPs, where sustained engagement of beneficiaries was a challenge, especially for the longest programmes, e.g., participation in 10–12 sessions, due to mobility, family and caregiving obligations, and hunger [26]. Among parents and broader community members, time constraints, competing priorities, and logistical challenges limit their participation in contextual-level interventions (51). Much has been learned about the barriers in engaging, recruiting and retaining parents/caregivers [39, 40], to help improve their participation going forward.

We found few differences in the socio-demographic and socio-economic profiles between the girls invited to participate in DREAMS and those not invited. Related to this, the fact that high proportions of EAG were invited to DREAMS by 2018 (77%) indicates that many EAG met the targeting criteria. In part, this reflects the high vulnerability of young people in these settings [15]. The Girl Roster tool used to identify participants found many ‘eligible’ girls and IPs supplemented the Roster with other methods (e.g., consulting community-based organisations) to identify girls with greatest need for DREAMS. It was clear that many, if not most of the girls in these informal settlement areas could benefit from DREAMS interventions, posing a challenge for targeting (with limited resources) and a tension with communities promoting social justice and inclusion over exclusivity. A goal in such high-burden settings may be to offer the primary (priority) interventions universally while targeting specific groups of EAG for the secondary interventions like HIV testing.

Sustained participation in DREAMS interventions was not always equitable. We found that EAG who had experienced verbal violence (although the evidence was weak), or ever been in relationships (although numbers were relatively low) accessed fewer primary interventions than their fellow participants. There remains scope to strengthen EAG participation in the primary interventions through active involvement and engagement of DREAMS beneficiaries, and through continued use of dedicated mentors [41]. Also, findings from qualitative research with DREAMS mentors shows that many of the girls were hungry during their time in safe spaces (social asset building), and this could have impacted their participation, particularly in the longer-term curriculum-based interventions as noted above [42]. It is imperative that programs targeting highly vulnerable EAG, especially in the context of social vulnerabilities like food insecurity, identify mechanisms to counter these challenges and support girls’ continued participation. Provision of food and beverages could facilitate ongoing and active participation and enjoyment of the programme.

One key strength of this study was the high cohort retention rate, limiting the introduction of selection bias over time. Interview questions captured components of each DREAMS intervention allowing for detailed assessment of combinations of interventions among EAG. We utilised self-reported invitation to participate in DREAMS as a marker of who was a DREAMS beneficiary. This may have resulted in misclassification of some of the participants in cases where some EAG did not know whether they had been invited to DREAMS. However, using data across multiple years strengthened this measure, in that responses in each year were complemented by responses in subsequent years. Our definition of uptake as participation in at least one session may have over-estimated exposure to interventions comprised of multiple sessions. However, only few of the interventions were offered as a curriculum of multiple sessions, so it is unlikely to largely inflate exposure to DREAMS. In addition, half of the girls participated in three or more of the intended sessions (except for parenting programs), and so we expect them to have benefited from this exposure, in comparison to those who were never invited to DREAMS.

## Conclusions

As DREAMS investments continue and expand to new districts and countries, with a particular focus on early adolescent girls, it is important to understand whether and how such a complex intervention can reach the youngest adolescents. We found that it is possible to deliver, scale up, and sustain an ambitious multi-sectoral intervention among EAG in challenging urban contexts, with uptake and layering improving over time. Provision of a coherent primary package of integrated interventions reached the majority of EAG in two informal settlement areas of Nairobi. Many girls also accessed secondary interventions, although longer-term engagement of the most vulnerable EAG and their families can be enhanced with support like food provision. There also remains a need to expand access to contextual, community-based programs so that parents, partners and communities can support the healthy development of EAG. Given the importance of engaging early adolescents in interventions for HIV prevention, and the limited evidence on programming among EAG, findings from this study can help programme implementers and funders to strengthen the implementation and reach of complex interventions like DREAMS.

## Supplementary Information

**Additional file 1.** Quantitative questionnaire (English).

**Additional file 2. **Characteristics of Early Adolescent Girls (EAG) enrolled in the cohort study in 02017, compared to those retained and interviewed in 2019. ^¥^is the number in each category; ** Pearson chi-square tests, Fishers exact used when expected cell counts < 5, the *p*-value compares those followed-up vs those lost to follow-up.

**Additional file 3.** Awareness of DREAMS interventions in each year among EAG followed up in 2019. *Some of the participants seen in 2019 were not seen in 2018; **Invitation to DREAMS is defined cumulatively from 2017 up to the year indicated in each column.

**Additional file 4.** Uptake* of each intervention by round of interview. *Participation in the intervention *in the last 12 months* prior to date of interview; N*-The denominator for 2018 is those seen in 2018, as some individuals seen in 2019 were not seen in 2018.

**Additional file 5.** Layering of primary interventions: number of EAG who accessed each primary intervention and its combinations cumulatively by 2019 (N* = 456). *Number who accessed at least one primary intervention by 2019.

**Additional file 6.** Baseline cohort profile, cumulative invitation to participate and uptake of interventions (primary, secondary and contextual) cumulatively by 2019.

**Additional file 7.** Layering of individual and contextual level interventions: number of EAG who accessed at least one intervention across individual and contextual levels cumulatively by 2019 (N* = 485). *Number who accessed at least one intervention from any level by 2019.

## Data Availability

Data underlying published results will be accessible and open, subject to a transition period (available from the London School of Hygiene and Tropical Medicine data repository https://datacompass.lshtm.ac.uk by contacting researchdatamanagement@lshtm.ac.uk), as per the Open Access Policy of the Bill & Melinda Gates Foundation.

## Abbreviations

AGYW: Adolescent girls and young women
EAG: Early Adolescent Girls
DREAMS: Determined, Resilient, Empowered, AIDS-free, Mentored, and Safe lives
GEAS: Global Early Adolescent Study
IP: Implementing Partners
NUHDSS: Nairobi Urban Health and Demographic Surveillance System
PEPFAR: President’s Emergency Fund for AIDS Relief
OR: Odds Ratio
CI: Confidence Interval
