# Supplementary material for: Reaching early adolescents with a complex intervention for HIV prevention: findings from a cohort study to evaluate DREAMS in two informal settlements in Nairobi, Kenya
Source: BMC Public Health. 2021 Jun 10;21:1107. doi: 10.1186/s12889-021-11017-y (PMC8194171; doi:10.1186/s12889-021-11017-y)
Supplement: Supplementary file 1 — Additional file 1. Quantitative questionnaire (English). [file 12889_2021_11017_MOESM1_ESM.docx]

**Additional file 1: DREAMS Impact Evaluation quantitative questionnaire (10-14 year olds), Nairobi - Kenya (English)**

This survey will be administered to adolescent girls who were aged 10-14 years at 2017 baseline/round 1 who are selected to participate in the DREAMS Cohort Survey

| A.1. *FIELD WORKER'S CODE*   \|  \|  \|  \|  \|  \|  \|  \|  \| \| --- \| --- \| --- \| --- \| --- \| --- \| --- \| --- \|   A.2. Site   1. Korogocho 2. Viwandani   A.3. STRUCTURE ID   \|  \|  \|  \|  \|  \|  \|  \|  \|  \|  \|  \| \| --- \| --- \| --- \| --- \| --- \| --- \| --- \| --- \| --- \| --- \| --- \|   A.4. HOUSEHOLD ID   \|  \|  \|  \|  \|  \|  \|  \|  \|  \|  \|  \| \| --- \| --- \| --- \| --- \| --- \| --- \| --- \| --- \| --- \| --- \| --- \|   A.5. HOUSEHOLD HEAD NAME _______________________________________  A.6. RESPONDENT’S ID   \|  \|  \|  \|  \|  \|  \|  \|  \|  \|  \|  \|  \|  \|  \| \| --- \| --- \| --- \| --- \| --- \| --- \| --- \| --- \| --- \| --- \| --- \| --- \| --- \| --- \|   A.7. RESPONDENT’S NAME_______________________________________  A.8. RESPONDENT’S GENDER   1. Female   A.9. Interview visits   \| Interview visits \| \| \| \| \| \| --- \| --- \| --- \| --- \| --- \| \|  \| 1 \| 2 \| 3 \| Final visit \| \| Date  Interviewer name  Interview result \|  \|  \|  \|  \| \| Next visit: Date  Time \|  \|  \|  \| Total No.  of visits \|  \| \|  \|  \|  \|  \|  \|   A.9. RESULT OF INTERVIEW   1. Complete 2. Incomplete 3. Absent for extended period 4. Out-migrated 5. Refused 6. Structure located but whereabouts of respondent unknown 7. Parent/guardian refused 8. Dead 9. Incapacitated 10. Not age eligible 11. Other (specify) ______________________   A.9a. Survey   1. 2017 Baseline 2. 2019 GEAS Follow-up two   A10: CONSENT  A.10a. Household consent obtained?   1. No 2. Yes   A.10a1. DATE WHEN HOUSEHOLD CONSENT WAS OBTAINED:  A.10b. Individual assent obtained?   1. No 2. Yes   A.10b1. DATE WHEN INDIVIDUAL ASSENT WAS OBTAINED:  A.11 CONTACT INFORMATION [FOR COHORT RESPONDENTS]  We would like to get your contacts, do you have a cellphone?   1. No 2. Yes   A.12. RECORD CELLPHONE NUMBER   \|  \|  \|  \|  \|  \|  \|  \|  \|  \|  \| \| --- \| --- \| --- \| --- \| --- \| --- \| --- \| --- \| --- \| --- \|   99. Refused to give phone number  A.13. Do you have an alternate phone number that you can share in case we need to contact you in future?   1. No 2. Yes   A.14. RECORD ALTERNATE PHONE NUMBER   \|  \|  \|  \|  \|  \|  \|  \|  \|  \|  \| \| --- \| --- \| --- \| --- \| --- \| --- \| --- \| --- \| --- \| --- \|   A.15. RECORD RELATIONSHIP OF PERSON WITH ALTERNATE NUMBER   1. Self 2. Spouse 3. Bio daughter/son 4. Step/adopt daughter/son 5. Bio mother/father 6. Step mother/father 7. Bio sister/brother 8. Step sister/brother 9. Maternal uncle/aunt 10. Paternal uncle/aunt 11. Maternal grandparent 12. Paternal grandparent 13. Cousin 14. Niece/nephew 15. Brother in-law 16. Sister in-law 17. Mother/father in-law 18. Spouse's other wife 19. Other relative (specify) 20. Other non-relative (specify)   A.16 RESPONDENT’S RELATIONSHIP TO HOUSEHOLD HEAD   1. Self 2. Spouse 3. Bio daughter/son 4. Step/adopt daughter/son 5. Bio mother/father 6. Step mother/father 7. Bio sister/brother 8. Step sister/brother 9. Maternal uncle/aunt 10. Paternal uncle/aunt 11. Maternal grandparent 12. Paternal grandparent 13. Cousin 14. Niece/nephew 15. Brother in-law 16. Sister in-law 17. Mother/father in-law 18. Spouse's other wife 19. Other relative 20. Other non-relative   A.16a NUMBER OF PEOPLE CURRENTLY LIVING IN YOUR HOUSEHOLD  A.17 ENTER INTERVIEW DATE:  A.18 ENTER INTERVIEW START TIME [24 HOUR CLOCK]: |
| --- | --- | --- | --- | --- | --- | --- | --- | --- | --- | --- | --- | --- | --- | --- | --- | --- | --- | --- | --- | --- | --- | --- | --- | --- | --- | --- | --- | --- | --- | --- | --- | --- | --- | --- | --- | --- | --- | --- | --- | --- | --- | --- | --- | --- | --- | --- | --- | --- | --- | --- | --- | --- | --- | --- | --- | --- | --- | --- | --- | --- | --- | --- | --- | --- | --- | --- | --- | --- | --- | --- | --- | --- | --- | --- | --- | --- | --- | --- | --- | --- | --- | --- | --- | --- | --- | --- | --- | --- | --- | --- |
|  |

**SOCIODEMOGRAPHICS**

Intro. This survey is being asked of both boys and girls, some your age, some younger, some older, and in many different countries and cultures. Some of the items will apply to you, while others may not.

IA1. How old are you?

________ years old RECORD AGE IN YEARS

IA1a. When were you born? (Ask respondent to give the best estimate if they do not know the year)

RECORD 98 IF DAY IS NOT KNOWN

RECORD 998 IF MONTH IS NOT KNOWN

RECORD 9898 IF YEAR IS NOT KNOWN

IA3. Are you currently enrolled in school?

1. No
2. Yes
3. It is currently holiday break or vacation; otherwise, I would be going to school

996. Refuse to answer

IA4. Including you, how many people typically sleep in the same room as you?

1. I sleep alone
2. 1 other person
3. 2 or 3 other people
4. 4 or more other people

996. Refuse to answer

IA5. What is your race/ethnicity?

1. Kikuyu
2. Luo
3. Luhya
4. Kamba
5. Kisii
6. Kalenjin
7. Somali
8. Borana
9. Garre
10. Other__________________(Specify)
11. Embu/Mbeere
12. Meru
13. Maasai
14. Mijikenda/Digo/Rabai/Giriama/Duruma
15. Taita
16. Taveta
17. Teso
18. Kuria
19. Swahili
20. Burji

996. Refuse to answer

IA5A. Were you born in (name of site)?

1. No
2. Yes

998. Don't know

996. Refuse to answer

*(→If Yes to IA5A (born in site), skip to 1A6)*

*If No or don’t know to IA5A (born outside of site):*

IA5B. How old were you when you began living in (name of site)?

Age in years ___________

998. Don't know

996. Refuse to answer

IA5C. When you came to *(name of site)*, where did you come from?

1. Other slum in Nairobi
2. Other non-slum in Nairobi
3. Another city/urban area in Kenya
4. A rural area of Kenya________
5. Another country

999. Don't know

996. Refuse to answer

IA5Cother. Please type in the name of the rural area (County) in Kenya that you came from.

_________________________________________________

IA6. What is your religion?

*INTERVIEWER’S NOTE:*

- *Roman Catholic*
- *Protestants Churches- ACK, PCEA, Methodist, AIC, AIPCA, Lutheran*
- *Pentecostal/ Charismatic- Full gospel, Charismatic Catholics, PAG, KAG and other with similar characteristics*
- *Other Christians- Legio Maria, SDA, Jehovah Witness, Akorino etc.*

1. I do not have a religion
2. Catholic
3. Protestant
4. Pentecostal/Charismatic
5. Other Christian
6. Muslim
7. Other

998 Don’t know

996. Refuse to answer

*(→ If “I do not have a religion”, skip to IA9a*)

IA7. How important is religion to you?

1. Not important at all
2. Not very important
3. Somewhat important
4. Very important

996. Refuse to answer

IA8. In the past month, how often did you attend religious services (example: at a church, temple, or mosque)?

1. Never
2. Once this past month
3. Two or three times a month
4. Once a week or more

996. Refuse to answer

**MEASURING EXPERIENCES OF DREAMS**

1A9a. Have you heard of a program called ‘DREAMS’? DREAMS is an HIV prevention program targeting young girls and women that is being implemented by LVCT Health in Korogocho and Hope Worldwide in Viwandani

1. No → Skip to below matrix
2. Yes

1A9b From where / how did you hear about DREAMS?

1. Mass media (TV, radio, newspaper)
2. Social media (web, Facebook, phone apps, email)
3. Word of mouth (a friend, relative, neighbour)
4. School
5. A health facility
6. A community-based or non-governmental organisation
7. A government agency
8. Community health workers
9. Other: _______________________________

1A9c Have you been invited to participate in any DREAMS activity or service?

1. No → Skip to below matrix
2. Yes

998. Don't know

996. Refuse to answer

1A9ci [IF 1A9c =YES] Have you ever enrolled into/registered for DREAMS?

1. No
2. Yes

996. Refuse to answer

1A9cii [IF 1A9ci =YES] When did you enroll /register?

Month ________

Year ___________

1A9d What experience(s) have you had with DREAMS?

*(Keep this question open-ended, to hear the range of experiences as expressed in respondents own words, before probing for specific experiences in the matrix below).*

1A9e Do you know your DREAMS ID number identifier/Unique Identification Number (UIN)? It could be a reference number, a card or badge, or code

1. No
2. Yes

996. Refuse to answer

1A9f (If 1A9e =Yes) Are you willing to share your DREAMS ID with us? We would like this number/identifier so that we can look at your use of DREAMS interventions. All of the information that you give us will be kept confidential.

1. No → Skip to below matrix
2. Yes, ID Number _________________________________
3. Yes, but I don’t know the number

998. Don't know

996. Refuse to answer

1A9g [For those who said Yes to 1A9f] Field staff to record the source of the girl’s DREAMS ID number

1. Memory
2. Presented on a document/card/badge
3. Others (specify)

[Field staff can use these to complete 1A9cii] *This is to facilitate links with monitoring data*

**DREAMS Exposure Matrix**

| Age/Sex of participants to be asked in the DSS | Type of service or program in the DREAMS core package | a. Are you aware of the following service / program?   1. Yes 2. No   IF NO SKIP TO “g” | b. Have you ever used (participated in) the service / intervention?   1. No 2. Yes   998. Don't know  996. Refused  IF NO SKIP TO “g” | If ever used or participated in the intervention: | | | | | | | g. If never used: why not?   1. Didn’t need it 2. Haven’t heard of it 3. Not available to me   96. Other  98. Don't know  96. Refused | | |  |
| --- | --- | --- | --- | --- | --- | --- | --- | --- | --- | --- | --- | --- | --- | --- |
|  |  |  |  | b1. What was the name of the program? | c. Did you use/ participate in the service in the past 12 months?   1. No 2. Yes   998. Don't know  996. Refused  IF NO GO TO NEXT SERVICE | | d. How many sessions/times did you participate in? | ONLY ASKED IF THE RESPONSE IN IA9a is ‘Yes’  e. When you used the service in the last 12 months, was it a ‘DREAMS’ program?   1. No 2. Yes   998. Don't know  996. Refused | | f. Where / from which organization was it provided? |  |  |  |  |
| AGYW | 1A9g: Safe Spaces program for girls/young women |  |  |  |  | |  |  | |  |  | | |  |
| AGYW and males in the DSS | 1A9h: HIV testing and counselling services at a health facility, mobile clinic, home, community, self-testing or safe space |  |  |  |  | |  |  | |  |  | | |  |
|  | IA9i: Partner testing (HIV testing with your sexual partner) |  |  |  |  | |  |  | |  |  | | |  |
|  | IA9j: Linkage to ART treatment |  |  |  |  | |  |  | |  |  | | |  |
| AGYW | IA9k:Condom provision at health facilities or in community |  | Have you ever received condoms at a health facility or in the community? |  | Did you receive condoms from a health facility or in the community in the last 12 months? | |  | When you received the condoms in the last 12 months, was it from a ‘DREAMS’ program? | |  |  | | |  |
| AGYW | IA9k1: Condom promotion, education and demonstration |  |  |  |  | |  |  | |  |  | | |  |
| AGYW | IA9l: Counselling on, and provision of contraception / family planning |  | Have you ever received Counselling on and provision of contraception / family planning? |  | Did you receive Counselling on and provision of contraception / family planning in the last 12 months? | |  | When you received the Counselling on and provision of contraception / family planning in the last 12 months, was it from a ‘DREAMS’ program? | |  |  | | |  |
| AGYW | IA9m Post-violence care counselling and services (e.g., HIV and STI testing, or linkage with the legal system |  |  |  |  | |  |  | |  |  | | |  |
| AGYW | 1A9n: HIV and STI testing services after an experience of violence (including sexual, physical or emotional violence) |  |  |  |  | |  |  | |  |  | | |  |
| AGYW | 1A9o: Any other post-violence care services (e.g., legal or police) |  |  |  |  | |  |  | |  |  | | |  |
| AGYW | IA9p: Post-exposure prophylaxis (PEP) (Drugs that can be taken to prevent HIV infection after possible exposure to the virus, including post-violence) |  |  |  |  | |  |  | |  |  | | |  |
| AGYW | IA9p1: Pre-exposure prophylaxis (PrEP) (Drugs that can be taken to prevent HIV infection *before* possible exposure to the virus) |  |  |  |  | |  |  | |  |  | | |  |
|  | *School-based Activities* | | | | | | | | | | | | |  |
| AGYW (in school) | IA9q: School-based HIV education/programs, such as life skills, Health Choices; Shuga; Tupange; My Health My Choice; violence prevention & gender norms |  |  |  |  | |  |  | |  |  | | |  |
|  | *Exposure to: Social Protection Interventions* | | | | | | | | | | | |  |  |
| AGYW | IA9r: Cash transfer to family / household *or AGYW* |  |  |  | |  |  | |  |  | |  | | |
| AGYW | IA9s: Educational subsidies (e.g., support for school fees, uniforms, books, or stationery) to help girls stay in school |  |  |  | |  |  | |  |  | |  | | |
| AGYW | IA9t: Microfinance program |  |  |  | |  |  | |  |  | |  | | |
| AGYW | IA9u: Financial literacy training for girls/young women |  |  |  | |  |  | |  |  | |  | | |
| AGYW | IA9v: Savings group/table banking |  |  |  | |  |  | |  |  | |  | | |
| AGYW | IA9w: vocational/business skills training |  |  |  | |  |  | |  |  | |  | | |
| All | IA9x: Violence prevention-related training or education in the community such as SASA, gender norms training |  |  |  | |  |  | |  |  | |  | | |
| AGYW | IA9y: Parent/care-giver ‘positive caregiving’ program – Family Matters! |  |  |  | |  |  | |  |  | |  | | |
| All | IA9z Have you heard of /participated in of any other program designed to encourage healthy, HIV-free living?  IA9z_a1: What program(s) have you heard of or participated in? |  |  |  | |  |  | |  |  | |  | | |
| AGYW | IA9za_a: Ajiri dada (by AMURT) |  |  |  | |  |  | |  |  | |  | | |
| AGYW | IA9zb_a:Tackle Africa for adolescent men in Korogocho /Viwandani (by MYSA) |  |  |  | |  |  | |  |  | |  | | |
| AGYW | IA9zc_a: Ushahidi |  |  |  | |  |  | |  |  | |  | | |
| AGYW | IA9zd_a: Wezesha dada |  |  |  | |  |  | |  |  | |  | | |

**FAMILY**

**Structure**

IIA1. Who is the person who most looks after you/takes care of you? This is sometimes called your primary or main caretaker (Choose 1)

1. There is no one who looks after me
2. Mother
3. Father
4. Step-mother
5. Step-father
6. Brother/ Step brother
7. Sister/ Step sister
8. Grandmother
9. Grandfather
10. Aunt
11. Uncle
12. Other adult family member (specify)
13. Other adult non-family member (specify)
14. Other__________
15. Refuse to answer

IIA1_other11 What other adult family member takes care of you?

_________________________________________________

IIA1_other12 What other adult non-family member takes care of you?

_________________________________________________

IIA1_other997 What other person takes care of you?

_________________________________________________

IIA1b [If IIA1= Other adult family member, other adult non-family member, other] Is your main caretaker male or female?

1. Male
2. Female

IIA2bro. How many brothers do you have? This includes step-brothers as well as those who do not live with you.

NUMERICAL

1. None *(→ If 0 (zero) to IIA2bro (no brothers), skip to IIA2sis)*

999. Don’t know

996. Refused

IIA2bro. How many live with you at home?

NUMERICAL (Cannot be greater than IIA2bro)

0. None

996. Refused

IIA2bro_old. How many of them are older than you?

NUMERICAL (Cannot be greater than IIA2bro)

1. None

999. Don’t know

996. Refused

IIA2sis. How many sisters do you have? This includes step-sisters as well as those who do not live with you.

NUMERICAL

1. None *(→ If 0 (zero) to IIA2sis (no sisters), skip to IIA3)*

999. Don’t know

996. Refused

IIA2sis. How many live with you at home?

NUMERICAL (Cannot be greater than IIA2sis)

0. None

996. Refused

IIA2sis_old. How many of them are older than you?

NUMERICAL (Cannot be greater than IIA2sis)

1. None

999. Don’t know

996. Refused

IIA3o. *[note]. We’d like to learn about things that happened to you over the last year.*

Which of the following things happened to you in the last year? [Select all that apply]

1 changed school

2 had a boyfriend or girlfriend

3 had a new sibling born

4 had a close friend or family member who died

5 got married or engaged

6 dropped out of school

7 went back to school after being out of school for at least one school year

8 had a parent who moved out

9 family member imprisoned or jailed

10 got a job

11 lost a job

12 had a parent/caregiver who became too sick to work or take care of the family

0 none of the above

996 Refuse

*(If IIA3b=4)*

IIA3. Have any members of your immediate family died, like a parent, or a brother or sister?

1. No (skip to IIA3c)
2. Yes

996. Refused

*(→ If No to IIA3 (no death in family), skip to* IIA3c*)*

*IIA3a. If yes to IIA3:* Who died? *(Select all that apply)*

1. Mother
2. Father
3. Brother or sister
4. Grandparent
5. Close friend
6. Someone else

996. Refused

*IIA3b. If father died (if IIA3a=2): How old were you when your father died?*

[Record age in years. If less than one year, record 00]

*IIA3bi. When did your father die?*

NUMERICAL

999. Don’t know

996. Refused

*IIA3c.* Is your father ill and perhaps needs caring for?

0. No

1. Yes

996. Refused

999. Don’t know

*IIA3d.* (If *IIA3c*=Yes) Are you involved in caring for your father?

1. Yes
2. No

996. Refused

999. Don’t know

IIA3e. If mother died (if IIA3a=1): How old were you when your mother died? (If don’t know, skip to IIA3f)

IIA3f. When did your mother die?

*IIA3g.* Is your mother ill and perhaps needs caring for?

0. No

1. Yes

996. Refused

999. Don’t know

*IIA3h.* (If *IIA3g*=Yes) Are you involved in caring for your mother?

1. Yes

2. No

996. Refused

**Family Connection/Communications**

IIB1 Who do you usually talk to if you have worries or concerns?

*(Pick the one you are most likely to talk to)*

1. Mother/main female caretaker
2. Father/main male caretaker
3. Brother
4. Sister
5. Friends
6. Grandparent
7. Other family member/relatives
8. Teacher
9. Someone at a school clinic, health center or youth center like a doctor, nurse or social worker

997. Other (specify________________)

1. I do not speak to anyone when I have questions or worries

IIB1_other Who would be another person you usually talk to if you have worries or concerns?

_________________________________________________

IIB2. How comfortable do you feel talking with [response IIA1] about:

IIB2a Things that worry you

IIB2b Changes with your body

IIB2c Problems with boyfriend or girlfriend

1. Not at all comfortable
2. Not very comfortable
3. Somewhat comfortable
4. Very comfortable

997. Not applicable (no boy-/girlfriend)

996. Refuse to answer

IIB3 Do you feel that [response IIA1] cares about what you are thinking and feeling?

1. Not at all
2. Not much
3. Somewhat
4. A lot

999. Don’t know

996. Refused

IIB4 Do you feel close to your mother/main female caretaker? *(By close, we mean that you can talk to that woman and tell her about personal and important things)*

1. Not at all
2. Not much
3. Somewhat
4. A lot
5. I have no mother or female caretaker

999. Don’t know

996. Refused

IIB5 Do you feel close to your father/main male caretaker? *(By close, we mean that you can talk that man about personal and important things)*

1. Not at all
2. Not much
3. Somewhat
4. A lot
5. I have no father or male caretaker

999. Don’t know

996. Refused

**Family Monitoring**

IIC. To what extent are these things true about [response IIA1]?

IIC1a Knows who my friends are by name

IIC1b [Item displayed only if in school, if IA3=1] Knows my grades/how I am doing in school

IIC1c Usually knows where I am

1. Not true at all
2. Not very true
3. Somewhat true
4. Very true

996. Refused

999. Don’t know

**II.C.A. Family Expectations**

IIC. To what extent are these things true about what [response IIA1] expects of you?

IIC2a [Item displayed only if in school, if IA3=1] Have good grades

IIC2b [Item displayed only if out of school, if IA3=0] Return to school

IIC2c Graduate from high school

IIC2d Go to university

1. Not true at all
2. Not very true
3. Somewhat true
4. Very true

996. Refused

999. Don’t know

IIC3. My [IIA1] approve(s) of me having a boyfriend/girlfriend at this time in my life.

Is this:

1. Not true at all
2. Not very true
3. Somewhat true
4. Very true

996. Refused

999. Don’t know

IIC4. When does [IIA1] expect you to marry?

1. After primary school
2. After secondary school
3. After I graduate from university or college
4. When I decide that I want to marry
5. They don’t expect me to marry

996. Refused

999. Don’t know

997. Other (Specify)

**PEERS**

**NUMBER OF CLOSE FRIENDS**

Now I will ask you a few questions about your friends, NOT counting the people in your family.

IIIA1. How many close friends (boys and/or girls) do you have? *(By close friends, I mean those that you can talk about feelings and share secrets.)*

IIIA1a Male friends

IIIA1b Female friends

1. None
2. 1
3. 2
4. 3
5. 4
6. 5
7. 6 or more

999. Don’t know

996. Refused

If “None” for both males and females, skip to section IV.

IIIB During a normal week, how often do you spend time with your close friends outside of school?

1. Never (no times per week)
2. Not very often (1 or 2 times a week)
3. Often (3-4 times a week)
4. Very often (nearly every day)

996. Refused

**III.C. PERCEIVED PEERNORMS (CLOSE FRIENDS)**

IIIC1. How many of your close friends think that it is important to

IIIC1a Attend school regularly

IIIC1b Study hard

IIIC1d Be good in sports

IIIC1e Be popular with people your age

IIIC1f Pay attention to their appearance

IIIC1g Have a boyfriend or girlfriend

IIIC1h Have sexual intercourse

1. None
2. Few
3. Most
4. All

999. Don’t know

996. Refused

IIIC2. In general, how many of your close friends do you think:

IIIC2a Smoke cigarettes (tobacco)

IIIC2b Drink alcohol (store bought or home brewed)

IIIC2c Use drugs

IIIC2d Have dropped out of school permanently

1. None
2. Few
3. Most
4. All

999. Don’t know

996. Refused

**SCHOOL: CONNECTEDNESS AND ASPIRATIONS**

(If not in school, IA3=0 skip to IVA10)

**School Connectedness**

IVA1. What grade or class are you in?

1. Primary Grade 1
2. Primary Grade 2
3. Primary Grade 3
4. Primary Grade 4
5. Primary Grade 5
6. Primary Grade 6
7. Primary Grade 7
8. Primary Grade 8
9. Secondary Form 1
10. Secondary Form 2
11. Secondary Form 3
12. Secondary Form 4
13. Vocational/trade school

999. Don’t know

996. Refused

IVA2.How much school do you think you will complete?

1. Leave before finishing primary school
2. Complete primary school
3. Complete secondary school
4. Complete university or college
5. Other (specify)

996. Refused

IVA3. Compared with others students in your class, how well do you think you are doing with your grades?

1. A lot worse
2. Worse
3. About the same
4. Better
5. A lot better

999. Don’t know

996. Refused

IVA4. Have you thought about dropping out of school this year?

1. Never
2. Yes, sometimes
3. Yes, a lot

999. Don’t know

996. Refused

IVA5. Do you feel that there is an adult (a teacher or someone else) in school who really cares about you as a person?

1. No, no one really cares
2. Yes, some of the time
3. Yes, all the time

999. Don’t know

996. Refused

IVA6. Do you feel that there is an adult (a teacher or someone else) in school who really cares if you do your work?

1. No, no one really cares
2. Yes, some of the time
3. Yes, all the time

999. Don’t know

996. Refused

IVA7. During the past month, how many days did you miss school for any reason except when school was closed or for holidays?

1. None
2. 1-2 days
3. 3-5 days
4. More than 5 days

999. Don’t know

996. Refused

*(→ If None or Don’t know or refused to answer to IVA7 (did not miss any days), skip to IV.B1.)*

IVA8. [If yes IVA7=1, 2 or 3 (missed school)] What were the main reasons you missed school last month? (Check all that apply)

1. Sick
2. Lack of school fees
3. [Only valid if 1A2=1] Having my period
4. Help out at home e.g because of a sick relative
5. Babysit younger brothers/sisters
6. Work to earn money
7. Hang out with friends
8. Studying for exam
9. To attend family events e.g. funeral/burial
10. School indiscipline e.g. not doing homework

997. Other______ (specify)

999. Don’t know

996. Refused

IVA8a_other. Please specify what those other reasons were for missing school.

___________________________________________________

**IV.A.10 Out of school**

*(→ If in school, IA3=1, skip to IVB1)*

Some young people who are participating in this study are not in school right now, or have never been in school. If that is true for you we would like to make sure we include your thoughts.

IVA9. Have you ever been in school?

1. No
2. Yes

999. Don’t know/not sure

996. Refused

*(→ If No to IVA9, skip to IVA12 - reasons for why not in school)*

IVA10. If Yes to IVA9. What is the highest grade or class you completed?

1. Primary Grade 1
2. Primary Grade 2
3. Primary Grade 3
4. Primary Grade 4
5. Primary Grade 5
6. Primary Grade 6
7. Primary Grade 7
8. Primary Grade 8
9. Secondary Form 1
10. Secondary Form 2
11. Secondary Form 3
12. Secondary Form 4
13. Vocational/trade school

999. Don’t know/not sure

996. Refused

IVA11.How long has it been since you left school?

1. Less than 1 year
2. Between 1 to 2 years
3. Between 2 to 3 years
4. More than 3 years

IVA12. What are some of the main reasons you are not in school? *(Check all that apply)*

1. Lack of school fees, uniforms, materials
2. Got pregnant
3. Got married
4. Sickness
5. Needed/wanted to earn money
6. Not a good student/failed in school
7. Not interested

997. Other______ (specify)

999. Don’t know

996. Refused

IVA12_other Please specify the other reason you are not in school.

___________________________________________________

**School Structure**

*(→ If out of school IA3*=0 *skip to* VA1*)*

IVB1. Which best describes the students in your school? They are…

1. All girls
2. All boys
3. Both boys and girls

996. Refused

IVB2. What kind of school do you go to?

1. Public school
2. Private, non-religious or secular school
3. Religious school

997. Other type of school______ (specify)

999. Don’t know

996. Refused

IVB2_other. Please specify the other type of school you go to.

________________________________________

IVB3. Which best describes the teachers in your school?

1. Mostly women (very few or no men)
2. Mostly men (very few or no women)
3. Both men and women

996. Refused

IVB4. All schools are different. Which of the following are available for students to use at your school?

IVB4.a. Toilets or latrines with doors?

IVB4.b. Running water

IVB4.c. Soap

IVB4.d. Computers

IVB4.e. Sports or other clubs

1. No
2. Yes

999. Don’t know/not sure

996. Refused

**Neighborhood**

**Cohesion**

VA1. The following questions are about adults in your neighborhood. That is, people who live in the same area, but are not your family or relatives. Tell me how much you think that the following is true.

VA1a People in my neighborhood look out for and help their neighbors

VA1b People in my neighborhood can be trusted

VA1c People in my neighborhood know who I am

VA1d People in my neighborhood care about me

1. Not true at all
2. Not very true
3. Somewhat true
4. Very true

999. Don’t know

996. Refused

**Perceived Social Control**

VA2. How likely is it that an adult in your neighborhood would do something like intervene if children or teenagers were?

VA2a Damaging property

VA2b Spraying paint on walls (graffiti)

VA2c Bullying or threatening another person

VA2d Fighting with another person

VA2e Doing something illegal in the neighborhood like selling drugs

1. Very unlikely
2. Not very likely
3. Somewhat likely
4. Very likely

999. Don’t know

996. Refused

**Neighborhood Safety**

VA3 How much do you think that the following are true about safety in your neighborhood?

VA3a There are a lot of safe places where people my age can spend time outdoors in my neighborhood

VA3b Every few weeks, an adolescent or adult in my neighborhood gets beaten up or mugged

VA3c Every few weeks, some adult in my neighborhood gets beaten up or mugged

VA3d I have seen people using or selling drugs in my neighborhood

VA3e In the morning or later in the day, I have often seen drunk people on the streets in my neighborhood

VA3f Most adults in my neighborhood respect the law

VA3g I feel safe when I walk in my neighborhood during the day

VA3h People in my neighborhood often damage or steal each other’s property

VA3i I feel safe walking in my neighborhood by myself at night

VA3j Boys or girls are often harassed or called names in my neighborhood

1. Not true at all
2. Not very true
3. Somewhat true
4. Very true

999. Don’t know

996. Refused

VA4a. Sometimes children feel unsafe or threatened when they are in their neighborhood, on the way to

school, or in school. For example, afraid of being attacked, bullied or being hurt. Has this happened

to you in the last year?

1. Never
2. Rarely
3. Sometimes
4. Often

999. Don’t know

996. Refused

VA4b. [If not in school, IA3=no] Sometimes children feel unsafe or threatened when they are in their

neighborhood. For example, afraid of being attacked, bullied or being hurt. Has this happened to you

in the last year?

1. Never
2. Rarely
3. Sometimes
4. Often

999. Don’t know

996. Refused

*(→If never or don’t know to VA4a or VA4b (do not feel unsafe), skip to VA8)*

VA5. If VA4a is not ‘never’, Can you tell me where you feel unsafe or threatened? *(Select all that apply)*

VA5_1 In your neighborhood

VA5_2 On the way to school

VA5_3 In your classroom

VA5_4 On the playground, gym, or sports field in school

1. No
2. Yes

999. Don’t know

996. Refused

VA6. If Yes to VA5_1 or VA5_2: Who or what makes you feel unsafe or threatened in your neighborhood?

*(Select all that apply)*

1. Adults
2. Boys or girls your age
3. Other things (specify)
4. Motorcycle and car accidents
5. Animals such as dogs

996. Refused

VA6_other Please specify what makes you feel unsafe or threatened.

________________________________________

VA7. If Yes to VA5_3 or VA5_4: Who or what makes you feel unsafe or threatened in school? *(Select all that*

*apply)*

1. Teachers or other adults
2. Classmates or other students
3. Other things (example: dogs, other animals, car accidents)

996. Refused

VA7_other Please specify what makes you feel unsafe or threatened.

________________________________________

VA8. Is there a person who you would go to if you felt threatened or unsafe?

1. No
2. Yes

999. Don’t know

996. Refused

*(→ If No to VA8 (no one they would go to), skip to VA10)*

VA9. Who would you go to for help or advice if you felt unsafe or threatened? *(Select all that apply)*

1. Parent/caretaker
2. Sibling
3. Other family member/relatives
4. Friend
5. Boyfriend/girlfriend
6. Teacher or coach
7. Religious leader
8. Police
9. Neighbour

997. Other____ (specify)

996. Refused

VA9_other Please specify the other person you would go to for help or advice if you felt unsafe or threatened.

________________________________________

VA10 Do you ever carry a weapon like a gun, razor, knife, stone or a club for protection?

1. Never
2. Rarely
3. Sometimes
4. Often

999. Don’t know

996. Refused

VA11 Have you ever lived on the streets or been homeless?

0. No [SKIP TO V1f1]

1. Yes

996. Refused [SKIP TO V1f1]

VA11a. If VA11 =Yes: Have you ever lived on the streets or been homeless in the past 12 months?

0. No

1. Yes

996. Refused

***Vignettes***

*I am now going to present a few made-up stories about some boys and girls.  I am then going to ask you what you think the people in the stories might do or what you think you would do if it was happening to you*

*FEMALE version*

Questions V1f1 to V5f10 are only asked to girls

*Alice is in your grade. She is attracted to Joseph, who is in the same grade, but she doesn’t know him and has never spoken with him in person. Most of her friends say they have boyfriends but she has never had one before. She wants to get his attention, but is not sure how.*

1. What do you think she is most likely to do?
2. Ask a friend to tell *Joseph* that Allice likes him
3. Pass him a note
4. Go up and talk to him directly

4. Nothing, just wait hoping she will meet him

1. What about you? What do you think you would do in that situation?
2. Ask a friend to tell *Joseph* that you like him
3. Pass *Joseph*a note
4. Go up and talk to *Joseph* directly
5. Nothing, just wait hoping you will meet him

*Alice decides to talk to Joseph herself. She is walking out of school with her friends when they run into a group of boys that includes Joseph. They all start talking about a friend’s party that is happening next Friday. Alice wants to know if Joseph is going, but is afraid to ask.*

1. What do you think she is most likely to do?
2. Ask if any of the boys are going to the party
3. Ask *Joseph* directly if he is going to the party
4. Get a friend to ask *Joseph* if he is going to the party
5. Say nothing and hope that someone else will ask *Joseph* if he is going
6. Ask *Joseph*’s friend if he knows if *Joseph* is going to the party
7. What do you think you would do if you were in that situation?
8. Ask if any of the boys are going to the party
9. Ask *Joseph* directly if he is going to the party
10. Get a friend to ask *Joseph* if he is going to the party
11. Say nothing and hope that someone else will ask A if he is going
12. Ask *Joseph*’s friend if he knows if *Joseph* is going to the party

*It is Friday, and Alice and Joseph are both at the party with their friends. Alice sees that Joseph is standing in a corner across the room. What would it take for her to talk to him?*

1. She would talk to him if:
2. Her friends challenged or encouraged her to go up to *Joseph*
3. *Joseph* was alone
4. *Joseph* came up to her or noticed her in another way
5. She knew from a friend that *Joseph* liked her
6. She would not talk to *Joseph* under any circumstance
7. As it turns out one of *Joseph*’s friends tells Alice that he likes her. Knowing this, what do you think she will do?
8. Go up and speak with *Joseph* directly
9. Do nothing and hope *Joseph* will notice her
10. Give *Joseph* “a look”
11. Walk by and bump into *Joseph* “accidentally”
12. Ignore *Joseph*
13. As it turns out Alice’s friends tell her that it is inappropriate for someone her age to be interested in boys. What do you think Alice will do?
14. Go up and speak with *Joseph* directly
15. Do nothing and hope *Joseph* will notice him
16. Give *Joseph* “a look”
17. Walk by and bump into *Joseph* “accidentally”
18. Ignore *Joseph*
19. If you were in Alice’s situation and you knew that *Joseph* liked you, what do you think you would do?
20. Go up and speak with *Joseph* directly
21. Do nothing and hope *Joseph* will notice me
22. Give *Joseph* “a look”
23. Walk by and bump into *Joseph* “accidentally”
24. Ignore *Joseph*

*It is also quite possible that Alice might not talk with Joseph at all.*

1. What is the most likely reason that she might *not* talk to him?
2. She is too embarrassed/shy
3. She is afraid of his rejection
4. She would be teased by his friends if she did so
5. She worries about being scolded because she has been told to stay away from boys
6. There is no reason why Alice would not talk to *Joseph*
7. If you were in the same situation, why might you not talk to him?
8. I would be too embarrassed/shy
9. I would be afraid of rejection by the boy
10. I would be teased by my friends
11. I would be afraid because I am not allowed to have a boyfriend
12. I can’t think of a reason why I would not talk to him

*Alice and Joseph did talk. They exchanged phone numbers, and Alice is now back home and thinks back on her evening.*

1. How is she feeling? (Pick the one that best describes her feeling.)
2. Happy
3. Proud
4. Nervous
5. Unsure
6. Afraid

**VIGNETTE #2: TEASING/ BULLYING**

*FEMALE Version*

*Ever since she was a child, Jane has always felt bored playing with other girls, and prefers to play with boys. She is now 13 and one day after school she sees her male classmates standing in a circle chatting in the middle of the playground. Jane wants to join them. She approaches them asking to join in.*

1. What do you think her male classmates do when *Jane* asks to join in?
2. They welcome her to join, just like anyone else
3. They say that she can’t join since the conversation is for boys only
4. They refuse and call her names for wanting to talk about boys stuff
5. They allow her to join because she is harmless
6. They allow her to join because she is good at the games they play
7. They allow her to join, but tease her

*The boys refuse to allow Jane to join their conversation.*

1. Why do you think they refuse to let her join their circle?
2. Because they feel uncomfortable discussing boy stuff with a girl
3. Because they think girls should hang out with girls and boys with boy
4. Because they think that *Jane* is very weird/unusual
5. Because they don’t want to be friends with someone they think is gay/homosexual

*Conversation turns to football. Again, Jane asks to join. Again she is told no.*

1. What do most of *Jane*’s female classmates think about her not being allowed to join the boys’ game?
2. They think she should be able to participate
3. They think it is unfair, but girls are never allowed to play with boys
4. They think she is weird and just makes trouble for herself
5. They think she is probably gay/homosexual

*Since Jane is not allowed to join the boys, she tries to join the girls’ group instead.*

1. What do you think the other girls will do?
2. They welcome her, just like anyone else
3. They let her join the group, but ignore her
4. They say that she can’t join because she is not a real girl
5. They allow her to join them but tease her
6. What are most of *Jane*’s female classmates most likely to think of her for wanting to join boys’ conversations and activities?
7. They admire her for acting as she wishes
8. They think that *Jane* is weird
9. They disapprove of *Jane*’s behavior
10. They think that *Jane* is probably gay/homosexual
11. What do you think you would do if you were with your classmates and *Jane* wanted to join?
12. Welcome her, just like anyone else
13. Let her join the group, but ignore her
14. Say that she can’t join because she is not a real girl
15. Allow her to join but make fun of her
16. All the girls and boys refuse to play with *Jane*. How do you think she feels?
17. She does not really care
18. She is confused and does not understand why she cannot play
19. She is angry that nobody likes her for who she is
20. She is sad and feels rejected
21. She is scared that something is wrong about her
22. How do you think *Jane*’s *mother* reacts to the fact that *Jane* prefers to play with boys?
23. Her mother tells *Jane* that she needs to start behaving “like a girl”
24. Her mother encourages *Jane* to be who she wants to be
25. Her mother is embarrassed by *Jane*
26. Her mother worries what the neighbors will think
27. Her mother takes her to a doctor/local healer/pastor
28. Her mother doesn’t care one way or another about *Jane’*s behavior
29. How do you think *Jane*’s *father* reacts to the fact that *Jane* prefers to play with boys?
30. Her father says that *Jane* needs to start acting “like a girl”
31. Her father encourages *Jane* to be who she wants to be
32. Her father is embarrassed by *Jane*
33. Her father worries what the neighbors will think
34. Her father takes her to a doctor/local healer/pastor
35. Her father doesn’t care one way or another about *Jane’*s behavior

**VIGNETTE #3: PUBERTY**

*Mary is 15 years old. She has been worried for a long time that all the other girls in her group were becoming curvier and starting to develop breasts. Until recently, Mary had seen none of those changes herself. The other day she got her first period; and she has started noticing hair where she didn’t have it before.*

1. What is *Mary* most likely to feel about the body changes she is experiencing, and the fact that she is going through puberty?
2. She is happy that she is becoming a grownup
3. She thinks that she is sick and something is terribly wrong
4. She is embarrassed about the changes she is experiencing
5. She is worried about the change
6. She is confused and wants more information about the changes she is experiencing

*Mary is confused about the changes that she is experiencing.*

1. What is she most likely to do next?
2. Tell no one that she has finally started puberty
3. Speak with someone about her concerns
4. Search for information without talking to anyone
5. Try to hide her body changes

*Mary tells her mother about her body changes.*

1. How is her mother most likely to *first* react to the fact that *Mary* has finally begun puberty?
2. Her mother tells *Mary* she is happy now that she is becoming a woman
3. Her mother makes fun of her
4. Her mother tells her that now that she is growing up, it is time to take on more responsibilities at home
5. Her mother tells her she should no longer play with boys
6. Her mother will teach her about hygiene and about the meaning of periods

*For years, Mary has been the subject of jokes and teasing by her more mature girlfriends. Now they start seeing that Mary is also maturing.*

1. What do you think they are most likely to do?
2. They will make fun of her for being slow
3. They will be too embarrassed to say anything
4. They will tell her now that she is a woman it is time to get a boyfriend
5. They will see it as normal and pay no attention to it

*Mary’s friends tease her for being slower than everyone else to enter puberty, and continue to tease her over the next few weeks. She is feeling more and more alone. She decides to speak with her older sister.*

1. What is her sister most likely to say to her?
2. “Ignore them”
3. “You are a woman now; go find a boyfriend”
4. “You need to stay at home more”
5. “You should be proud that you are growing up”
6. Her sister probably won’t say anything

**VIGNETTE#4: BOYS WHO PREFER TO PLAY WITH GIRLS**

*Ever since he was a child, Charles has always felt bored playing with other boys, and prefers to play with girls. He is now 13, and one day after school he sees his female classmates standing in a circle chatting in the middle of the playground. Charles wants to join them. He approaches them asking to join in.*

1. What do you think the girls would do when *Charles* asks to join in?
2. They welcome him to join, just like anyone else
3. They say that he can’t join since the conversation is for girls only
4. They refuse and tease him for wanting to act like a girl
5. They allow him to join because she isn’t boy-ish
6. They allow him to join, but tease him because he is a boy
7. They allow him to join, but tease him because he acts like a girl

*The girls refuse to allow Charles to join their conversation.*

1. If you were with these girls, would you agree with their decision?
2. Yes because he would have caused trouble in the group
3. Yes because he is weird and you prefer keeping him at a distance
4. Yes because he would have been bullied if he had joined
5. No, you would have let him join like anyone else
6. No, you would have let him join but would have kept him at a distance
7. Why do you think the girls would refuse to let him join their circle?
8. Because they feel uncomfortable discussing girl stuff with a boy
9. Because they think girls should hang out with girls and boys with boys
10. Because they think that Charles is very weird/unusual
11. Because they don’t want to be friends with someone they think is gay/homosexual

**VIGNETTE#5: PREGNANCY**

*Mercy is 14 years old and in Form 1. Her boyfriend, Andrew, is also 14 years old. Recently, Mercy realized that she is pregnant, and told Andrew that he had made her pregnant. The next day, Mercy’s best friend notices that she is not herself and asks her what the problem is.*

1. How do you think *Mercy* is *feeling*? *(Pick the one best choice)*
2. Scared
3. Happy
4. Proud
5. Angry
6. Sad
7. How do you think *Mercy* is most likely to react to realizing that she is pregnant?
8. Angry that Andrew got her pregnant
9. Deny that she is pregnant
10. Accuse Andrew of trapping her into being a mother
11. Accepts the pregnancy but refuses any further involvement with Andrew
12. Depends on how she feels about Andrew
13. How do you think you would react if you were ever in *Mercy*’s situation?
14. You would hope that the problem would go away on its own and would do nothing
15. You would be happy to have a baby with Andrew
16. You would be happy to have a baby but would not want Andrew involved
17. You would not want to have a baby with Andrew
18. You would not want a baby with anyone at this time in your life

*Mercy tells her friend that she is pregnant and that Andrew has caused the pregnancy.*

1. What is her friend most likely to advise *Mercy* to do?
2. Run away from home
3. Have the baby and raise it
4. Have the baby and give it up for adoption
5. Assume equal responsibility with Andrew and make a joint decision
6. Get an abortion
7. Do whatever Andrew decides

*Mercy is scared and plans to run away from home. She tells her younger sister. Her younger sister tells their parents that Mercy is pregnant and Andrew is responsible.*

1. How will *Mercy*‘s parents react when they find out that their daughter is pregnant? They will…
2. Accuse Andrew of tricking *Mercy* into having sex
3. Kick *Mercy* out of the house
4. Say that they will find the money for Mercy to have an abortion
5. Say they will force *Mercy* to marry Andrew as soon as possible
6. Say they will take care of the baby no matter what *Mercy* decides to do with Andrew

*Mercy’s parents insist that their daughter is not responsible for the pregnancy. They say that Mercy is too young and “innocent” to be having sex, and accuse Andrew of tricking their daughter into having sex with him.*

1. What will *Mercy* do next?
2. She denies responsibility
3. She decides to end the relationship with Andrew after the baby is born
4. She marries Andrew as soon as she can
5. She runs away from home
6. She does nothing and just waits to see what happens next

*Mercy thinks she should end the pregnancy and finds a place where she can have an abortion at a good price.*

1. What is *Mercy* most likely to do next?
2. Continue the pregnancy/keep the baby
3. End her relationship with Andrew and continue the pregnancy alone
4. Ask Andrew for help getting money for an abortion, but deal with it alone
5. Ask Andrew to accompany her to have the abortion

*If Mercy decides she wants to continue the pregnancy…*

1. What will she do next?
2. Tell her parents and go away to have the baby secretly
3. Go away to have the baby secretly without telling her family
4. Stay and have the baby with her family’s support
5. Stay and have the baby with Andrew’s support
6. What do *you* think *Mercy* *should* do in this situation? What would be the right thing to do?
7. Continue the pregnancy/keep the baby
8. End her relationship with Andrew and continue the pregnancy alone
9. Ask Andrew for help getting money for an abortion, but deal with it alone
10. Ask Andrew to accompany her to have the abortion
11. What do you think *your friends would do* if they ever were in this kind of situation?
12. Continue the pregnancy/keep the baby
13. End her relationship with Andrew and continue the pregnancy alone
14. Ask Andrew for help getting money for an abortion, but deal with it alone
15. Ask Andrew to accompany her to have the abortion

**Media**

*The following questions are about your access to and use of media, for example: TV, radio, movies,*

*computers, Internet, mobile phones.*

VIA1. For each item, please tell me if you have access to it.

VIA1a Television

VIA1b Radio

VIA1c Computer/laptop /tablet (eg. iPad) with internet connection

VIA1d Cell/mobile phone

VIA1f Social media account such as Facebook, Twitter, etc.

1. No, do not have
2. Yes, but don’t have my own
3. Yes, I have my own

997. Other ______ (specify)

996. Refused

VIA2. [If in school (IA3=1)] On a typical school day, how many hours in total do you spend watching

TV/movies, playing computer or video games, using the Internet, chatting to friends online or on a mobile

phone, or using other media?

1. None (I do not use electronic media)
2. About 1 hour or less
3. About 2 hours
4. About 3 hours
5. Between 4 and 5 hours
6. More than 5 hours

996. Refuse to answer

VIA2a. If VIA1f is yes (1 or 2). Do you use the internet or social media to get information about: *(Select all)*

1 Puberty

2 Romantic relationships /dating relationships

3 Sex

4 Pregnancy

5 Contraception

6 Sexually transmitted infections

8 Periods/cycle

9 Abortion

0 None

VIA3. [Skip if IIIa1a=0 and IIIA1b=0; have no friends] How often do you do the following?

VIA3a Contact your friends using texting or other social media

VIA3b Talk to your friends directly by phone or computer (for example, using a phone or video call)

1. Never (never includes “do not have a phone”)
2. Less than weekly
3. Weekly
4. Daily

996. Refuse to answer

VIA3c Have you ever made friends with someone through the internet?

1 Yes

0 No

997 Don’t know

996 Refuse to answer

VIA4 Sometimes young people watch pornography, that is, movies or videos that show people’s genitals (private parts) during sexual scenes. Have you seen this type of program before?

1. Never
2. Occasionally
3. Yes, often
4. Yes, very often

996. Refuse to answer

**Normative romantic relations (NRR), sexual double standard (SDS), masculine sexual prowess scales (MSP)**

The following questions are about adolescents or people your age, for each statement, we would like to know how much YOU agree or disagree with each statement. We also want to know how much you think YOUR FRIENDS would agree or disagree with each statement.

The response options for each statements are:

1. Disagree a lot
2. Disagree a little
3. Neither agree nor disagree
4. Agree a little
5. Agree a lot

999. Don’t know

996. Refuse to answer

| Variable name |  | | Do YOU agree or disagree ? | Would MOST OF YOUR FRIENDS agree or disagree ? |
| --- | --- | --- | --- | --- |
| GN1 | SDS | A girl will lose interest in studying if she has a boyfriend |  |  |
| GN2 | NRR | A boy and a girl your age should be able to spend time together alone if they want to |  |  |
| GN3 | SDS | Girls your age often get into "trouble" when they have boyfriends |  |  |
| GN4 | NRR | A boy should be able to have a girlfriend if he wants to |  |  |
| GN5 | SDS  MSP | Boys have girlfriends for fun more than love |  |  |
| GN6 | NRR | It’s normal for a boy your age to want a girlfriend |  |  |
| GN7 | SDS | Girls who have boyfriends are irresponsible |  |  |
| GN8 | NRR | Boys should have girlfriends to discover love |  |  |
| GN9 | MSP | Boys like girls who wear revealing clothes |  |  |
| GN10 | NRR | A girl should be able to have a boyfriend if she wants to |  |  |
| GN11 | SDS | Girls are the victims of rumors if they have boyfriends |  |  |
| GN12 | SDS | Boys tell girls they love them when they don't |  |  |
| GN13 | NRR | A boy should have more than one girlfriend to gain experience |  |  |
| GN14 | SDS | Adolescent girls should avoid boys because they trick them into having sex |  |  |
| GN15 | SDS  MSP | Boys have girlfriends to show off to their friends |  |  |
| GN16 | NRR | A girl should have more than one boyfriend to gain experience |  |  |
| GN17 | MSP | Boys generally compete for the prettiest girls |  |  |
| GN18 | NRR | A girl can have a boyfriend as long as she continues working well in school |  |  |
| GN19 | SDS | Boys feel they should have girlfriends because their friends do |  |  |
| GN20 | NRR | A boy can have a girlfriend as long as he continues working well in school |  |  |
| GN21 | SDS  MSP | Adolescent boys lose interest in a girl after they have sex with her |  |  |
| GN22 | NRR | It’s normal for a girl to want a boyfriend at your age |  |  |
| GN23 | SDS  MSP | Adolescent boys fool girls into having sex |  |  |

**VII- Health literacy/information**

VIIA1. [If answered no at baseline) Have you heard of HIV/AIDS?

1. No
2. Yes

999. Don’t know

996. Refuse to answer

VIIA2. [If answered no at baseline) Have you heard about condoms that men can put on before having sexual intercourse? By sexual intercourse we mean when a man puts his penis in a woman’s vagina

1. No
2. Yes

999. Don’t know

996. Refuse to answer

VIIA3. [if VIIA2=1] Have you seen a condom?

1. No
2. Yes

999. Don’t know

996. Refuse to answer

VIIA4 Here are some statements about pregnancy and HIV. Please tell me whether you think the statement is true, or false, or whether you don't know.

VIIA4_1 A girl can get pregnant the first time that she has sexual intercourse.

VIIA4_2 A boy/girl can get HIV the first time he/she has sexual intercourse.

VIIA4_3 A girl can get pregnant after kissing or hugging.

VIIA4_4 A girl can swallow a pill every day to protect against pregnancy.

VIIA4_5 Using a condom can protect against pregnancy.

VIIA4_6 Using a condom can protect against HIV.

VIIA4_7 You can get HIV through kissing.

VIIA4_8 A girl can have a shot or injection that will protect against pregnancy.

VIIA4_9 A girl or boy can swallow a pill before having sex that will protect against HIV.

VIIA4_10 A girl can use herbs to prevent a pregnancy.

VIIA4_11 A boy can get a girl pregnant before he has his first ejaculation

VIIA4_12 A boy can be fertile every day of the month

VIIA4_13 It’s normal for a girl to have periods that don’t come at the same time each month?

VIIA4_14 A girl or boy can take a pill every day to protect against HIV

1. False
2. True

999. Don’t know

996. Refuse to answer

VIIA5. If an adolescent girl in your neighborhood needed contraception (birth control), do you think she would know where to get it?

1. No
2. Maybe
3. Yes

997. Don't know what it is/don’t understand

999. Don’t know

996. Refuse to answer

VIIA6 The following questions are about your knowledge or experience using health services. Tell me how much you think that the following is true.

VIIA6a I know where to go if I am sick

VIIA6b I know where to go to get condoms

VIIA6c I know where to go if I needed to get information about menstrual periods

VIIA6d I know where to go if I needed to get contraception (birth control)

VIIA6e I would feel too shy or embarrassed to go to a clinic, hospital or pharmacist if I needed contraception (birth control)

VIIA6f I would feel too shy or embarrassed to go get a condom if I needed it

VIIA6g I think I would be treated well if I were to go to a clinic, hospital or pharmacist for contraception

VIIA6h I know where to go to get treatment for a sexually transmitted infection

1. No
2. yes

997. Don't know what it is/don’t understand

999. Don’t know

996. Refuse to answer

VIIB (If age>14): The following questions are about your knowledge of family planning methods:

Have you ever heard about?

VIIB_a female sterilization? PROBE: Women can have an operation to avoid having any more children.

VIIB_b Male sterilization PROBE: Men can have an operation to avoid having any more children.

VIIB_c implant? PROBE: Women can have one or several small rods placed in their upper arm by a doctor or nurse, which can prevent pregnancy for one or more years.

VIIB_d IUD/PPIUD? PROBE: Women can have a loop or coil placed inside them by a doctor or a nurse

VIIB_e injectables? PROBE: Women can have an injection by a health provider that stops them from becoming pregnant for one or more months

VIIB_f Daily pill? PROBE: Women can take a pill every day to avoid becoming pregnant.

VIIB_g emergency contraception? PROBE: As an emergency measure after unprotected sexual intercourse women can take special pills at any time within five days to prevent pregnancy

VIIB_h standard days method or Cycle Beads? PROBE: A Woman can use a string of colored beads to know the days she can get pregnant. On the days she can get pregnant, she and her partner use a condom or do not have sexual intercourse.

VIIB_i Lactational Amenorrhea Method or LAM? PROBE: For the first six months after birth a woman who is breastfeeding may be able to avoid pregnancy if her periods have not returned.

VIIB_j rhythm method? PROBE: Women can avoid pregnancy by not having sexual intercourse on the days of the month they think they can get pregnant

VIIB_k withdrawal method? PROBE: Men can be careful and pull out before climax.

VIIB_l any other ways or methods that women or men can use to avoid pregnancy?

0 No

1 Yes

996 refuse

For each where VIIB1a-l ==1

VIIB2 Did you receive information about these methods from [select all that apply]

1 parent

2 sibling

3 other family member

4 friend

5 school

6 healthcare provider

7 TV, the radio, the internet, or books

If any of VIIB1a-l ==1

VIIB3: Have you ever used any of these methods to avoid pregnancy with a partner?

0 No

1 Yes

996 refuse

If VIIB3=1

VIIB3a Which methods have you used? [tick all that apply]

1. Female sterilization.
2. Male sterilization
3. Implant
4. IUD/PPIUD
5. Injectables
6. Pill
7. Emergency Contraception
8. Male Condom/Nirodh
9. Female Condom
10. Std. Days/Cycle beads
11. LAM
12. Rhythm method
13. Withdrawal
14. Other traditional methods

996. refused to answer

VIIB3b: (If age>14) For the following questions, please tell me if you agree or not with the following statements:

VIIB3ba Contraception is only for married women.

VIIB3bb With contraception, a young couple can love each other with peace of mind.

VIIB3bc Adolescents or young women who use contraception are seen as promiscuous

VIIB3bd Practicing family planning allows young women to prepare for having children

VIIB3be Using any form of modern contraception can cause infertility

- - - 1. Agree a lot
      2. Agree a little
      3. Neither agree, nor disagree
      4. Disagree a little
      5. Disagree a lot

996. Refuse to answer

**VIII.A. ADOLESCENT HEALTH**

You are doing really great so far. Now we are going to talk about your health and your body.

VIIIA1. In general, how is your health?

1. Poor
2. Fair
3. Good
4. Excellent

999. Don't know

996. Refuse to answer

VIIIA2. How much do you weigh *(kgs)*

Kgs___

999. Don't know

996. Refuse to answer

*(→ If weight is known, record self-reported weight and also request in VIIIA2a to take actual weight)*

*(→ If Don’t know or Refuse to answer, skip to VIIIA2a. and request to take actual weight)*

VIIIA2a. RECORD ACTUAL WEIGHT MEASURED

__________kgs

999. Refused to be measured

VIIIA3. What is your height? (Select feet and inches or centimeters)?

Feet and inches___

Cm___

999. Don't know

996. Refuse to answer

*(→ If height is known, record self-reported height and also request in VIIIA3a to take actual height)*

*(→ If Don’t know or Refuse to answer, skip to VIIIA3a. and request to take actual height)*

VIIIA3a. RECORD ACTUAL HEIGHT MEASURED

__________

999. Refused to be measured

VIII4. How do you think of yourself in terms of your weight?

1. Much too thin
2. A bit too thin
3. About the right weight
4. A bit too fat
5. Much too fat

999. Don't know

996. Refuse to answer

VIIIA5. How do you think of yourself in terms of your height?

1. Much too tall
2. A bit too tall
3. About the right height
4. A bit too short
5. Much too short

999. Don't know

996. Refuse to answer

VIIIA6 As children grow up, their bodies start to change. Thinking about your body, how fast do you feel you are maturing/changing compared with other girls your age?

1. Faster
2. About the same
3. Slower

999. Don't know

996. Refuse to answer

**Pubertal Maturation (GIRLS ONLY):**

VIIIB1. (If no, don’t know or refused at baseline) Have your breasts started to grow/become larger?

1. No
2. Yes

999. Don’t know

996. Refuse to answer

VIIIB2. (If no, don’t know or refused at baseline) Have you started to have periods?

1. No
2. Yes

999. Don’t know what periods are

996. Refuse to answer

*(→ If No or Don’t know to VIIIB2 (did not get period), skip to section VIII.D)*

VIIIB2a. If Yes to VIIIB2 (got period): How old were you when you first got your period?

NUMERICAL RESPONSE [Limit 7-16 years old]

999. Don't know/ can’t remember

996. Refuse to answer

*If the girls answered no period or don’t know what periods are* VIIIB2=1 or 999*: skip to* VIIID1

VIIIB3. (if girls answered yes to period at baseline or at midline) Tell me how much you think the following

statements are true for you.

VIIIB3a I feel ashamed of my body when I have my period

VIIIB3b Having a period tells me I am a woman

VIIIB3c It’s important that I keep my period secret from anyone

VIIIB3d I feel proud that I have my periods

VIIIB3e Getting my period is not a big deal for me

1. Not true at all
2. Not very true
3. Somewhat true
4. Very true

999. Don't know

996. Refuse to answer

*Only ask if the girls answered yes to having periods (VIIIB2=1)*

VIIIB4 Do you usually track your menstrual cycle?

1. No
2. Yes

999. Don't know

996. Refuse to answer

VIIIB5 Do you usually know when you will get your period?

1. No
2. Yes

999. Don't know

996. Refuse to answer

VIIIB6 The last time that you had your period…

VIIIB6_1 What sanitary products did you use to manage your last period? (Multiple responses allowed)

1. Tampon
2. Disposable sanitary pad
3. Reusable sanitary pad
4. Menstrual cup
5. Toilet paper
6. Cotton wool
7. Mattress
8. Pieces of cloth
9. Other (Specify)

98. Don't know

99. Refuse to answer

VIIIB6_1 Other. What other product did you use to manage your last period?

_________________________________________________

VIIIB6_2 Did you miss school because of your period?

1. No
2. Yes

999. Don't know

996. Refuse to answer

VIIIB6_3 The last time you missed school because of your period was it because?

(select all that apply)

1 Pain

2 There was nothing to manage my period at school

3 Tradition or beliefs

4 Teasing

**VIII.D. Body Comfort**

VIIID1. Boys/girls have different feelings about their bodies and the changes they experience. Here are some statements about how you feel about your body. Please tell me how much you agree or disagree with each.

VIIID1a On the whole, I am satisfied with my body

VIIID1b I worry about the way that my body looks

VIIID1c I like the way I look

VIIID1d I like looking at my body

VIIID1e I feel like I am beautiful

VIIID1f I often wish my body were different

VIIID1h I am worried that my body is not developing normally

1. Agree a lot
2. Agree a little
3. Neither agree, nor disagree
4. Disagree a little
5. Disagree a lot

996. Refuse to answer

(→ *If the respondent answered No to both breast change (VIIIB1) and periods (VIIIB2) skip to VIIID3)*

*VIIID2. If Yes to puberty/body changes:* During the teenage years, girls’ bodies change in many ways, at different times. I would like to know how you feel about some of these changes. Please tell me how much you agree or disagree with the following statements.

VIIID2a I like the fact that I am becoming an older woman

VIIID2b I like that response [IIA1] may treat me more like an adult now

VIIID2c In general, I am proud of the pubertal changes I am going through

1. Agree a lot
2. Agree a little
3. Neither agree, nor disagree
4. Disagree a little
5. Disagree a lot

996. Refuse to answer

VIIID3. Have you talked with anyone about the body changes that happen as boys and girls grow up?

1. No
2. Yes

999. Don't know

996. Refuse to answer

*(→ If No/Don’t know to VIIID3 (did not talk with anyone about puberty), skip to section VIIID4)*

VIIID3a. If Yes to VIIID3 (talked to someone regarding pubertal changes): Who did you talk to? *(You may*

*select more than one person)*

1. Mother/female caregiver
2. Father/male caregiver
3. Sister
4. Brother
5. Other family member and relatives (e.g grandparents)
6. Friend/peer
7. Doctor/nurse or other person at a health centre
8. School teacher
9. LVCT/Hope mentors/teachers

997. Other _______ (specify)

996. Refuse to answer

VIIID3a_other Please specify the other person you talked to about pubertal changes.

___________________________________

VIIID4. In the last 12 months, have you discussed the following topics with anyone?

VIIID4a Sexual relationships

VIIID4b Pregnancy and how it occurs

VIIID4c Contraception

VIIID4d HIV/AIDS

VIIID4x [Only ask if VIIIB2=1]: How to take care of yourself during your period

1. No
2. yes

999. Don't understand

997. I don’t understand the question

996. Refuse to answer

*(→ If No or can’t remember to all items in VIIID4 (did not talk about sex/pregnancy/ contraception/HIV), skip to section IX.)*

VIIID5a If yes to VIIID4a: sexual relationships: Who did you talk to about sexual relationships? *(You may select more than one person)*

1. Mother/female caregiver
2. Father/male caregiver
3. Sister
4. Brother
5. Other family member
6. Friend/peer
7. Doctor/nurse or other person at a health center
8. School teacher
9. LVCT/Hope worldwide mentors/teachers

997. Other _______ (specify)

996. Refuse to answer

VIIID5a_other Please specify the other person you talk to about sexual relationships.

________________________________________

VIIID5b If yes to VIIID4b: talked about pregnancy and how it occurs

Who did you talk to about pregnancy? *(You may select more than one person)*

1. Mother/female caregiver
2. Father/male caregiver
3. Sister
4. Brother
5. Other family member
6. Friend/peer
7. Doctor/nurse or other person at a health center
8. School teacher
9. LVCT/Hope worldwide mentors/teachers

997. Other _______ (specify)

996. Refuse to answer

VIIID5b_other Please specify the other person you talk to about pregnancy.

________________________________________

VIIID5c If yes to VIIID4c: talked about contraception

Who did you talk to about contraception? *(You may select more than one person)*

1. Mother/female caregiver
2. Father/male caregiver
3. Sister
4. Brother
5. Other family member
6. Friend/peer
7. Doctor/nurse or other person at a health center
8. School teacher
9. LVCT/Hope worldwide mentors/teachers

997. Other _______ (specify)

996. Refuse to answer

VIIID5c_other Please specify the other person you talk to about contraception.

________________________________________

VIIID5d If yes to VIIID4d: talked about HIV/AIDS

Who did you talk to about HIV/AIDS? *(You may select more than one person)*

1. Mother/female caregiver
2. Father/male caregiver
3. Sister
4. Brother
5. Other family member
6. Friend/peer
7. Doctor/nurse or other person at a health center
8. School teacher
9. LVCT/Hope worldwide mentors/teachers

997. Other _______ (specify)

996. Refuse to answer

VIIID5d_other Please specify the other person you talk to about HIV/AIDS.

________________________________________

VIIID5e If yes to VIIID4e: talked about your period

Who did you talk to about your period? *(You may select more than one person)*

1. Mother/female caregiver
2. Father/male caregiver
3. Sister
4. Brother
5. Other family member
6. Friend/peer
7. Doctor/nurse or other person at a health center
8. School teacher
9. LVCT/Hope worldwide mentors/teachers

997. Other _______ (specify)

996. Refuse to answer

VIIID5e_other Please specify the other person you talk to about your periods.

________________________________________

**Gender Norms Scales**

**Stereotypical views about toughness versus weakness**

The following questions are about adolescents or people your age, for each statement, we would like to know how much YOU agree or disagree with each statement. We also want to know how much you think YOUR FRIENDS would agree or disagree with each statement.

The response options for each statements are:

1. Disagree a lot
2. Disagree a little
3. Neither agree nor disagree
4. Agree a little
5. Agree a lot

999. Don’t know

996. Refuse to answer

| Variable name |  | Do YOU agree or disagree? | Would MOST OF YOUR FRIENDS agree or disagree? |
| --- | --- | --- | --- |
| GN24 | Girls should avoid playing sports with boys because they get hurt easily |  |  |
| GN25 | Boys should be raised tough so they can overcome any difficulty in life |  |  |
| GN26 | Girls should avoid raising their voice to be lady like |  |  |
| GN27 | Boys should always defend themselves even if it means fighting |  |  |
| GN28 | Girls are expected to be humble |  |  |
| GN29 | Girls should always fight back if boys try to take advantage of them |  |  |
| GN30 | Girls need their parents’ protection more than boys |  |  |
| GN31 | Boys should be able to show their feelings without fear of being teased |  |  |
| GN32 | Boys who behave like girls are considered weak |  |  |
| GN33 | It's important for boys to show they are tough |  |  |

**IX. MENTAL HEALTH**

During adolescence, we know that people your age often experience emotional ups and downs, for example feeling really happy one day and really sad another day. That is normal. Here we would like to better understand if you experience emotional lows a lot. Also, we want to know if you have had experiences that might have caused you to feel very sad or low.

**IX.A. Depression**

We would like to know a little about how you are feeling. Tell me how much you agree with the following statements:

IXA1a In general, I see myself as a happy person

IXA1b I blame myself when things go wrong

IXA1c I worry for no good reason

IXA1d I am so unhappy I can’t sleep at night

IXA1e I feel sad

IXA1f I am so unhappy I think of harming myself

1. Agree a lot
2. Agree a little
3. Neither agree, nor disagree
4. Disagree a little
5. Disagree a lot

996. Refuse to answer

**IX.B. Adverse Childhood Experiences**

Now we would like to ask whether as a child you ever experienced any of these things. You may not want to tell us, and that is OK, but the reason we are asking is that it will help us better understand who you are and what you have experienced.

IXB1: In the last 12 months

IXB1a Have you ever been scared or felt really bad because grown-ups called you names, said mean things to you, or said they didn’t want you?

IXB1b Have you ever been scared that your parents or other adults were going to hurt you badly (so that you were injured or killed)?

IXB1c Have you ever felt like you are not loved or cared about?

IXB1d Have you ever felt like you have no one that protects you?

IXB1e Has there ever been a time of your life when you were totally on your own and had to take care of yourself for more than a short time?

IXB1f Have your parents/guardian ever drank too much alcohol or used drugs so they came home and were really abusive to you or your family?

IXB1g Has there ever been a time when your family did not have enough food because they had no money?

IXB1h Have you ever seen your mom being hit, beaten or threatened?

IXB1i Have you ever seen your mother or father so sad that they couldn’t take care of you?

IXB1j Have any of your parents ever been in prison/jail?

IXB1k Has your family ever been forced to leave your home/house?

IXB1l Has an adult ever touched you in your private parts except when being bathed?

IXB1m Has an adult ever attempted or forced you to have sexual intercourse?

1. Never
2. Sometimes
3. Often

999. Don't know

996. Refuse to answer

**IX.C. Bullying and Gender Based Violence**

IXC1. During the last six months, have you seen any of your *male* peers *bully or threaten* someone? By bullying we mean making threats, spreading rumors against someone, attacking someone verbally or excluding someone from a group on purpose.

1. No, I have not seen them bully/threaten someone
2. Yes, I have seen them bully/threaten both boys and girls
3. Yes, I have seen them bully/threaten girls
4. Yes, I have seen them bully/threaten other boys

999. Don't know

996. Refuse to answer

IXC2. During the last six months, have you seen any of your *female* peers *bully or threaten* someone?

1. No, I have not seen them bully/threaten someone
2. Yes, I have seen them bully/threaten both boys and girls
3. Yes, I have seen them bully/threaten boys
4. Yes, I have seen them bully/threaten other girls

999. Don't know

996. Refuse to answer

IXC3. During the last six months, have you seen any of your *male* peers start a *physical fight* with someone?

1. No, I have not seen them start a fight against someone
2. Yes, I have seen them start a fight against both boys and girls
3. Yes, I have seen them start a fight against girls
4. Yes, I have seen them start a fight against other boys

999. Don't know

996. Refuse to answer

IXC4. During the last six months, have you seen any of your *female* peers start a *physical fight* with someone?

1. No, I have not seen them start a fight against someone
2. Yes, I have seen them start a fight against both boys and girls
3. Yes, I have seen them start a fight against boys
4. Yes, I have seen them start a fight against other girls

999. Don't know

996. Refuse to answer

IXC5. During the last six months, has someone touched you in a way that you did not want to be touched?

1. No
2. Yes

998. Can’t remember

999. Don’t know

996. Refuse to answer

IXC6. If Yes to IXC1 or IXC2 (witnessed bullying by male or female peer): The last time you saw peers bully or threaten someone, did you try to do something (told them to stop, or called for help)?

1. No
2. Yes

998. Can’t remember

999. Don’t know

996. Refuse to answer

IXC7. During the last six months, have you been teased or called names by someone?

1. No
2. Yes, by both boys and girls
3. Yes, by a boy
4. Yes, by a girl

998. Can’t remember

999. Don’t know

996. Refuse to answer

*(→ If No or can’t remember to* IXC7 *(no bullying victimization), skip to IXC10)*

If yes… IXC7 about teased or called names

IXC8. If you were teased or called names, do you think this was because:

IXC8a. You are a girl

IXC8d The person thought you were acting like a boy

1. No
2. Yes

998. Can’t remember

999. Don’t know

996. Refuse to answer

IXC9. If yes to IXC7 (bullying victimization): The last time you were bullied or threatened was this in person (face-to-face), or by using the Internet/social media?

1. In person/face-to-face
2. Internet/social media
3. Both in person and through the Internet/social media

999. Don’t know

996. Refuse to answer

IXC10. During the last 6 months have you ever been slapped, hit or otherwise been physically hurt by a boy or girl in a way that you did not want?

1. No
2. Yes, by both boys and girls
3. Yes, by a boy or boys
4. Yes, by a girl or girls

999. Don’t know

996. Refuse to answer

IXC11. During the last 6 months, have you bullied or threatened another boy or girl for any reason?

1. No
2. Yes, both boys and girls
3. Yes, a boy or boys
4. Yes, a girl or girls

999. Don’t know

996. Refuse to answer

IXC12. In the last 6 months, have you ever slapped, hit or otherwise physically hurt another boy or girl in a way that they did not want?

1. No
2. Yes, by both boys and girls
3. Yes, by a boy or boys
4. Yes, by a girl or girls

999. Don’t know

996. Refuse to answer

**IX.D. Alcohol and Substance Use**

IXD1. Have you ever drank alcohol (except for religious purposes)?

1. No
2. Yes

999. Don’t know

996. Refuse to answer

*(→ If No or don’t know to IXD1 (never used alcohol), skip to IXD4)*

IXD2. How old were you when you had your first drink of alcohol?

1. 8 or younger
2. 9 years old
3. 10 years old
4. 11 years old
5. 12 years old
6. 13 years old
7. 14 years old
8. 15 years old
9. 16 years old

999. Don’t know/can’t remember

996. Refuse to answer

IXD3. In your lifetime, how often have you gotten drunk or very high from drinking alcohol?

1. Never
2. Once/ 1 time
3. 2-3 times
4. 4 or more times

999. Don’t know/can’t remember

996. Refuse to answer

IXD4. In your lifetime have you ever smoked cigarettes, a pipe, or chewed tobacco?

1. No
2. Yes

999. Don’t know

996. Refuse to answer

*(→ If No or* Don’t know *to* IXD4 *(never smoked/chewed tobacco), skip to* IXD6*)*

IXD5. If yes to [IXD4] (smoked/chewed): How many cigarettes do you normally smoke?

1. I do not smoke
2. 1 cigarette a week or less
3. Less than 1 cigarette a day
4. 2-5 cigarettes a day
5. More than 5 cigarettes a day

999. Don’t know

996. Refuse to answer

IXD6. Have you ever used (smoked or eaten) marijuana (grass, weed, pot, bhangi)?

1. No
2. Yes

999. Don’t know

996. Refuse to answer

IXD7. Have you ever used any other drugs that were not given to you to treat an illness? (These are sometimes referred to as “street drugs” such as Kuber, msi, jet fuel, cocaine, heroin, crack, mrambe, mandrax, miracle juice, petrol, glue/gum, injectables, chaves, taptap/maduya, mchele, miraa/muguka/khat

1. No
2. Yes

999. Don’t know

996. Refuse to answer

**Views about sexual behaviors and relationships**

The following questions are about adolescents or people your age, for each statement, we would like to know how much YOU agree or disagree with each statement. We also want to know how much you think YOUR FRIENDS would agree or disagree with each statement.

The response options for each statements are:

1. Disagree a lot
2. Disagree a little
3. Neither agree nor disagree
4. Agree a little
5. Agree a lot

999. Don’t know

996. Refuse to answer

| Variable name |  | Do YOU agree or disagree? | Would MOST OF YOUR FRIENDS agree or disagree? |
| --- | --- | --- | --- |
| GN34 | It is ok for an adolescent girl to have sex as long as she avoids getting pregnant |  |  |
| GN35 | In general, a girl should only have sex with someone she loves |  |  |
| GN36 | In general, if an adolescent girl says “no” to sex her boyfriend will dump her |  |  |
| GN37 | It is ok for an adolescent boy to have sex as long as he avoids getting a girl pregnant |  |  |
| GN38 | In general, a boy should only have sex with someone he loves |  |  |
| GN39 | It is okay to tease a girl who acts like a boy |  |  |
| GN40 | It’s the girl’s responsibility to prevent pregnancy |  |  |
| GN41 | Girls should be proud of their bodies as they become women |  |  |
| GN42 | It is okay to tease a boy who acts like a girl |  |  |
| GN43 | Boys and girls should be equally responsible for household chores |  |  |
| GN44 | A woman’s role is taking care of her home and family |  |  |
| GN45 | A man should have the final word about decisions in the home |  |  |
| GN46 | A woman should obey her husband in all matters |  |  |

**X.A. ROMANTIC RELATIONSHIPS**

Now we are interested in romantic relationships between young people your age in your community, and about your own experience with liking someone as more than just friends.

Sometimes two young people really like each other and try to spend private time together. Some people call this dating or having a boyfriend or girlfriend. This can happen between any two young people.

XA1. At what age do you think most girls in your community start having boyfriends?

NUMERICAL

999. Don’t know

996. Refuse to answer

XA2. At what age do you think most boys in your community start having girlfriends?

NUMERICAL

999. Don’t know

996. Refuse to answer

XA3. How important is it to you to have a girlfriend or boyfriend right now?

1. Not at all important
2. Not very important
3. Somewhat important
4. Very important

999. Don’t know

996. Refuse to answer

If XA3=2, 3 i.e. to have a boy or girl friend is very important or somewhat important go to XA4; otherwise skip to XA5

XA4. Why is it important for you to have a boy/girlfriend?

1. I want to be cared for
2. I want to be popular
3. I want to be loved
4. I want to be protected

997. Other (specify)

999. Don’t know

996. Refuse to answer

XA4e_other Please specify the other reason why having a boy/girlfriend is important

_______________________________________

XA5.Do you have *any close friends* your age who have had boyfriends or girlfriends?

1. No, I have no close friends
2. No, none of my close friends
3. Yes, some of my close friends
4. Yes, most of my close friends

999. Don’t know

996. Refuse to answer

XA6. Have you ever felt that you were in love with a boy or a girl?

1. No, neither
2. Yes, with a girl
3. Yes, with a boy
4. Yes, with both boys and girls

999. Don’t know

996. Refuse to answer

*(→ If No or don’t know to XA6 (never been in love), skip to XA11)*

If XA6=1 or 2, skip to XA8

If XA6=3, continue with XA7

XA7. The last time you were in love, was it with a boy or girl?

1. A boy
2. A girl

999. Don’t know

996. Refuse to answer

XA8. The last time you were in love, did this person like you back?

1. No
2. Yes

999. Don’t know

996. Refuse to answer

*(→ If No or Not sure to XA8 (did not like back), skip to XA11)*

XA9. Did you tell other people that he/she was your [girlfriend (if XA6=1 or *XA7=2*) OR boyfriend (if XA6=2 or *XA7=1*)]?

1. No
2. Yes

999. Don’t know

996. Refuse to answer

XA10. Did you ever have to keep the relationship secret from your [response IIA1]?

1. No
2. Yes

999. Don’t know

996. Refuse to answer

XA11. Today which statement best describes you? *(Select one)*

1. I have never been in a romantic relationship
2. I am not currently in a romantic relationship, but I have had a boy-/girlfriend in the past
3. I have more than one girlfriend
4. I have more than one boyfriend
5. I have a girlfriend
6. I have a boyfriend
7. I am engaged to be married to someone
8. I am married

999. Don’t know

996. Refuse to answer

*(→ If answer XA11=0* I have never been in a romantic relationship, *skip to XB9*

*If* in current relationships XA11=7,6,5,4,3,2

XA12_1 [If has a boyfriend *If* XA11=5 or 3] Can you talk to [response IIA1] about your boyfriend?

XA12_2 [If has a girlfriend *If* XA11=4 or 2] Can you talk to [response IIA1] about your girlfriend?

1. No
2. Yes

999. Don’t know

996. Refuse to answer

**XB. Current or Most Recent Boyfriend/Girlfriend**

The following questions are about your current or most recent relationship

If more than one boyfriend” XA11=3 or the same for “more than one girlfriend” XA11=2, Pick the one that is the most important for you.

XB1. Can you give me the initial (first letter) of this person?

ALPHABETICAL

999. Don’t know

996. Refuse to answer

XB2. How old is [response XB1]?

NUMERICAL

999. Don’t know

996. Refuse to answer

XB3a If current: XA11=7,6,5,4,3,2= Generally, how often do you spend time alone with [response XB1]?

XB3b If past: XA11=1 Generally, how often did you spend time alone with [response XB1]?

1. Never
2. Very rarely
3. Once a week
4. 2 times a week
5. 3-4 times a week
6. Every day

999. Don’t know

996. Refuse to answer

XB4 How much do you agree or disagree with the following statements about your relationship with [response XB1?] (If current: XA11=7,6,5,4,3,2)

XB4a_1 There are times when [response XB1] cannot be trusted

XB4a_2 [response XB1] makes me feel good about myself in a way my friends can't

XB4a_3 I sometimes do things because [response XB1] is doing them

XB4a_4 [response XB1] often influences what I do

XB4a_5 I sometimes do things because I don’t want to lose response XB1’s respect

XB4a_6 [response XB1] sometimes wants to control what I do

XB4a_7 Sometimes I don't know quite what to say to [response XB1]

XB4a_8 I would be uncomfortable having intimate conversations with [response XB1]

XB4a_9 Sometimes I find it hard to talk about my feelings with [response XB1]

XB4a_10 I feel comfortable talking with [response XB1] when I have a problem

XB4a_11 Sometimes I feel I need to watch what I say to [response XB1]

XB4a_12 [response XB1] cares about me

XB4a_13 I am very attracted to [response XB1]

XB4a_14 The sight of [response XB1] turns me on

XB4a_15 I would rather be with [response XB1] than anyone else

XB4a_16 [response XB1] always seems to be on my mind

XB4_17 [response XB1] and I are practically inseparable

If past: XA11=1

XB4b_1 There were times when X could not be trusted

XB4b_2 [response XB1] made me feel good about myself in a way my friends couldn’t

XB4b_3 I sometimes did things because [response XB1] was doing them

XB4b_4 [response XB1] often influenced what I did

XB4b_5 I sometimes did things because I didn’t want to lose [response XB1]’s respect

XB4b_6 [response XB1] sometimes wanted to control what I do

XB4b_7 Sometimes I didnt’ know quite what to say to [response XB1]

XB4b_8 I would be uncomfortable having intimate conversations with [response XB1]

XB4b_9 Sometimes I found it hard to talk about my feelings with [response XB1]

XB4b_10 I felt comfortable talking with [response XB1] when I had a problem

XB4b_11 Sometimes I felt I needed to watch what I said to [response XB1]

XB4b_12 [response XB1] cared about me

XB4b_13 I was very attracted to [response XB1]

XB4b_14 The sight of [response XB1] turned me on

XB4b_15 I would have rather been with [response XB1] than anyone else

XB4b_16 [response XB1] always seemed to be on my mind

XB4b_17 [response XB1] and I were practically inseparable

1. Agree a lot
2. Agree a little
3. Neither agree, nor disagree
4. Disagree a little
5. Disagree a lot

999 Don’t know

996 Refuse to answer

XB5 The following questions are about fights you may have had with response [XB1] in the last 12 months

XB5_1 Has [response XB1] ever thrown something at you in the last 12 months?

XB5_2 Has [response XB1] ever pushed, shoved, or grabbed you in the last 12 months?

XB5_3 Has [response XB1] ever slapped you in the face or head in the last 12 months?

XB5_4 Has [response XB1] ever hit you in the last 12 months?

1. No
2. Yes, one time
3. Yes, several times

999. Don’t know

996. Refuse to answer

XB6_1 Have you ever thrown something at [response XB1] in the last 12 months?

XB6_2 Have you ever pushed, shoved, or grabbed [response XB1] in the last 12 months?

XB6_3 Have you ever slapped response XB1] in the face or head in the last 12 months?

XB6_4 Have you ever hit [response XB1] in the last 12 months?

1. No
2. Yes, one time
3. Yes, several times

999. Don’t know

996. Refuse to answer

XB7. Have you ever told [response XB1]: “I love you” in the last 12 months?

1. No
2. Yes

999. Don’t know

996. Refuse to answer

XB8. Did [response XB1] ever tell you he/ she loved you in the last 12 months?

1. No
2. Yes

999. Don’t know

996. Refuse to answer

XB9. If XA11=0 or 1: Young people have different reasons for not having boyfriends or girlfriends.

What are the most important reasons that you do not have a boyfriend/girlfriend now? (You may select more than one reason)

1. I am too young
2. My parents/primary caregiver would be very angry
3. It would interfere with my work or school
4. I would get a bad reputation
5. It is against my culture/religion
6. I want to but do not have the opportunity
7. I am afraid of the consequences for my future
8. I don’t trust boys

999. Don’t know

996. Refuse to answer

997. Other (Specify)

**X.C. SEXUAL BEHAVIOR**

The following are questions about sexual behaviors. At times people your age do not understand the questions and at times they are uncomfortable. Whatever the reason, you should feel free not to answer any question or if you are embarrassed or uncomfortable with the questions being asked please feel free to stop. How much to answer is completely your choice and no one will criticize you for not wanting to go on.

First, we will ask some questions about what you think your close friends might have done together with someone as more than just friend, like someone they are in love with [e.g. boyfriend and girlfriend].

XC1. Have any of your close friends kissed with a boyfriend or girlfriend?

1. No, none
2. Yes, some
3. Yes, most

999. Don’t know

997. Don’t understand the question

996. Refuse to answer

XC2. Have any of your close friends touched another boy or girls’ private parts (e.g. breasts or genitals)?

1. No, none
2. Yes, some friends
3. Yes, many friends

999. Don’t know

997. Don’t understand the question

996. Refuse to answer

XC3. Have any of your close friends had sex (sexual intercourse)? This is when a man or boy puts his penis inside a girl’s or woman’s vagina.

1. No, none
2. Yes, some friends
3. Yes, many friends

999. Don’t know

997. Don’t understand the question

996. Refuse to answer

Now we would like to ask you about things that YOU might have done together with someone else as more than just friends (i.e. with a boyfriend/girlfriend). Remember that you can skip any question that you do not feel comfortable answering.

XC4. [If XB3a or XB3b is not 0,999,996, (has spent time alone with partner) automatically recode as “yes” + sex from XA11 and skip to XC5] Have you ever spent time alone with someone you were in love with in a private space without any adults around?

1. No
2. Yes with a boy
3. Yes with a girl

999. Don’t know

997. Don’t understand the question

996. Refuse to answer

XC5. Have you ever held hands with someone you were in love with?

1. No
2. Yes with a boy
3. Yes with a girl

999. Don’t know

997. Don’t understand the question

996. Refuse to answer

XC6. Have you ever hugged or cuddled with someone you were in love with? By this we mean when two young people hold each other close as more than just friends, to show love or affection.

1. No
2. Yes with a boy
3. Yes with a girl

999. Don’t know

997. Don’t understand the question

996. Refuse to answer

XC7. Have you ever kissed or been kissed by someone on the lips or with your tongue?

1. No
2. Yes with a boy
3. Yes with a girl

999. Don’t know

997. Don’t understand the question

996. Refuse to answer

XC8. Have you ever flirted with someone using a phone, email, or social media?

1. No
2. Yes with a boy
3. Yes with a girl

999. Don’t know

997. Don’t understand the question

996. Refuse to answer

XC9. [If XC5, XC6, XC7, or XC8 = 1 or 2] Have you ever sent a sexual picture of yourself to someone using the phone, email, or social media?

1. No
2. Yes, to a boy
3. Yes, to a girl

999. Don’t know

997. Don’t understand the question

996. Refuse to answer

XC10. [If XC2 different from “don’t understand”] Have you ever touched another boy or girls’ private parts or been touched by someone? By touching, we mean touching boys’ or girls’ private parts, breasts or other body parts in a sexual way.

1. No
2. Yes, a boy
3. Yes, a girl

999. Don’t know

997. Don’t understand the question

996. Refuse to answer

XC11. [If XC3 different from “don’t understand”] Have you ever had sexual intercourse?

1. No, never
2. Yes, one time
3. Yes, several times

999. Don’t know

997. Don’t understand the question

996. Refuse to answer

XC12. (If yes to any questions XC4 to XC11) Have you ever put your mouth on someone’s genitals (private parts), or has someone put their mouth on your genitals (private parts)? Some people call this oral sex.

1. No, never
2. Yes, one time
3. Yes, several times

999. Don’t know

997. Don’t understand the question

996. Refuse to answer

XC13. [If yes to any questions XC4 to XC11] Have you ever had anal sex? (This is when a man or boy puts his penis inside someone else's anus.)

1. No, never
2. Yes, one time
3. Yes, several times

999. Don’t know/can’t remember

997. Don’t understand the question

996. Refuse to answer

*NOTE TO INTERVIEWER: Software creates a grid based on the activities that the respondent says that he/she ever did. For each behavior, they are asked to list the age at first time. If interviewer administered, ask in relation to each behavior that the respondent indicated ’yes’ to).*

XC14. How old were you when you firs [*Insert activities below only  if ’yes’ earlier*]?

XC14a Spent time with someone in a private space without any adults around

XC14b Held hands

XC14c Cuddled/hugged

XC14d Kissed on the lips or with tongue

XC14e Flirted with someone using a phone or social media

XC14f Sent a sexual picture of yourself

XC14g Touched someone else’s genitals (private parts) in a sexual way

XC14h Had oral sex

XC14i Had sexual intercourse

XC14j Had anal sex

1. 7 or younger
2. 8
3. 9
4. 10
5. 11
6. 12
7. 13
8. 14
9. 15
10. 16

999. Don’t know

998. I never did that

996. Refuse to answer

*(→ Ask only if Yes to XC10 (touching private parts), else skip to XC16a)*

XC15a. The first time you either touched or were touched by someone (touching private parts, breasts or other parts of the body in a sexual way), what was your relationship to this person?

1. It was my boyfriend/girlfriend
2. A boy or girl in school or the community other than a boyfriend/girlfriend
3. It was my husband/wife
4. It was a stranger
5. It was a sex worker
6. It was my father or mother
7. It was my brother or sister
8. It was another relative
9. It was a teacher

997. Other _______ (specify)

996. Refuse to answer

XC15a_other Please specify the other person you touched or who touched you

________________________________________

XC15b. How old was this person?

1. Same age as me
2. Younger than me
3. 1-2 years older than me
4. 3-4 years older than me
5. 5 or more years older than me

999. Don’t know

996. Refuse to answer

XC15c. The first time you were either touched or were touched by someone, would you say you were willing, somewhat willing or not willing at all to? Willing means you gave permission or said it was OK, or that you did it because you wanted to and not because someone made you.

1. Very willing
2. Somewhat willing
3. Not really willing
4. Not willing at all

999. Don’t know

996. Refuse to answer

XC15d. Young people touch each other for different reasons. Please tell me which of the following statements best describes the first time you touched or were touched by someone.

XC15d_1 I wanted to show love

XC15d_2 I was curious about it

XC15d_3 My friends pressured me

XC15d_4 I felt obliged to my boyfriend/girlfriend

XC15d_5 I was threatened

XC15d_6 The person insisted and would not take “no” for an answer

XC15d_7 I was promised money or gifts

XC15d_8 I was physically forced

XC15d_9 I threatened or physically forced the person

XC15d_10 Other: ______________________

XC15d_11 I was given alcohol or drugs

1. No
2. Yes

999. Don’t know

996. Refuse to answer

XC15d_10_other Please specify the other reason why you touched or were touched by someone

________________________________________

*(→ Ask only if Yes to XC12 (oral sex), else skip to XC17a)*

XC16a. The first time you had oral sex with someone (put one’s mouth on someone’s genitals in sexual way or vice versa), what was your relationship to this person?

1. It was my boyfriend/girlfriend
2. A boy or girl in school or the community other than a boyfriend/girlfriend
3. It was my husband/wife
4. It was a stranger
5. It was a sex worker
6. It was my father or mother
7. It was my brother or sister
8. It was another relative
9. It was a teacher
10. I was paid

997. Other _______ (specify)

996. Refuse to answer

XC16a_other Please specify the other person you had oral sex with

________________________________________

*XC16*b How old was this person?

1. Same age as me
2. Younger than me
3. 1-2 years older than me
4. 3-4 years older than me
5. 5 or more years older than me

999. Don’t know

996. Refuse to answer

XC16c. *The first time you had oral sex with someone, would you say you were willing, somewhat willing or not willing at all to? Willing means you gave permission or said it was OK, or that you did it because you wanted to and not because someone made you.*

1. Very willing
2. Somewhat willing
3. Not really willing
4. Not willing at all

999. Don’t know

996. Refuse to answer

Young people have oral sex for different reasons. Please tell me which of the following statements best describes the first time you had oral sex with someone.

XC16d_1 I wanted to show love

XC16d_2 I was curious about it

XC16d_3 My friends pressured me

XC16d_4 I felt obliged to my boyfriend/girlfriend

XC16d_5 I was threatened

XC16d_6 The person insisted and would not take “no” for an answer

XC16d_7 I was promised money or gifts

XC16d_8 I was physically forced

XC16d_9 I threatened or physically forced the person

XC16d_10 I was given alcohol or drugs

XC16d_11 Other: ______________________

1. No
2. Yes

999. Don’t know

996. Refuse to answer

XC16d_11_other Please specify the other reason why you had oral sex

________________________________________

*(→ Ask only if Yes to XC11 (sexual intercourse), else skip to XI)*

XC17a. The first time you had sexual intercourse with someone, what was your relationship to this person?

1. It was my boyfriend/girlfriend
2. A boy or girl in school or the community other than a boyfriend/girlfriend
3. It was my husband/wife
4. It was a stranger
5. It was a sex worker
6. It was my father or mother
7. It was my brother or sister
8. It was another relative
9. It was a teacher

997. Other _______ (specify)

996. Refuse to answer

XC17a_other Please specify the other person you had sexual intercourse with

________________________________________

XC17b. How old was this person?

1. Same age as me
2. Younger than me
3. 1-2 years older than me
4. 3-4 years older than me
5. 5 or more years older than me

999. Don’t know

996. Refuse to answer

XC17c. The first time you had sexual intercourse with someone, would you say you were willing, somewhat willing or not willing at all to? Willing means you gave permission or said it was OK, or that you did it because you wanted to and not because someone made you.

1. Very willing
2. Somewhat willing
3. Not really willing
4. Not willing at all

999. Don’t know

996. Refuse to answer

Young people have sexual intercourse for different reasons. Please tell me which of the following statements best describes the first time you had sexual intercourse with someone.

XC17d_1 I wanted to show love

XC17d_2 I was curious about it

XC17d_3 My friends pressured me

XC17d_4 I felt obliged to my boyfriend/girlfriend

XC17d_5 I was threatened

XC17d_6 The person insisted and would not take “no” for an answer

XC17d_7 I was promised money or gifts

XC17d_8 I was physically forced

XC17d_9 I threatened or physically forced the person

XC17d_10 I was given alcohol or drugs

XC17d_11 Other: ______________________

1. No
2. Yes

999. Don’t know

996. Refuse to answer

XC17d_11_other Please specify the other reason why you had sexual intercourse

________________________________________

XC17e. The first time you had sexual intercourse, do you remember if you or your partner did anything to protect against pregnancy or sexually transmitted infections?

1. No
2. Yes

999. Don’t know

996. Refuse to answer

*(→ If No or Don’t know to* XC17e*, skip to* XC17g*)*

XC17f. [If yes to XC17e] what method(s) were used? *(Select all methods that apply)*

1. Male condom
2. Pill
3. Injection
4. Female condom
5. Foam/jelly
6. Periodic abstinence/rhythm
7. Withdrawal
8. Emergency contraception
9. Male sterilization
10. IUD/Coil
11. Implant

997. Other (specify)

996. Refuse to answer

XC17foth. [If XC17f=997 other] What other method did you use?

_________________________________________________________

XC17g. The first time you had sexual intercourse, were you under the influence of alcohol or drugs?

1. No
2. Yes

999. Don’t know

996. Refuse to answer

**XD: Sex with current or last partner**

Questions XD1 to XD7 asked only if respondent has ever had sex XC11=1 or 2, else skip to XI

Now we will ask some questions about what you have done together with [response XB1], your current or last boyfriend/girlfriend or partner.

XD1. Have you ever had sexual intercourse with [response XB1]?

1. No
2. Yes

999. Don’t know

996. Refuse to answer

XD2. [If XD1=1 (has had intercourse with [response XB1])] Do you feel that having sex with [response XB1] has led to a closer relationship between you two?

1. No
2. Yes

999. Don’t know

996. Refuse to answer

XD3 [If XD1=1 (has had intercourse with [response XB1])] Some people are worried about sexually transmitted diseases. How concerned are you about getting an STD from [response XB1]?

1. Not at all concerned.
2. Not really concerned
3. Somewhat concerned
4. Very concerned

997. Don’t know what this is

999. Don’t know

996. Refuse to answer

XD4[If XD1=1 (has had intercourse with [response XB1]) Some young people are worried about pregnancy. How concerned are you about getting pregnant with [response XB1]?

1. Not at all concerned.
2. Not really concerned
3. Somewhat concerned
4. Very concerned

999. Don’t know

996. Refuse to answer

XD5. [If XD1=1 (has had intercourse with [response XB1]) AND XA11=7,6,5,4,3,2 (has a current partner)] Are you and [response XB1] doing anything to avoid a pregnancy?

1. No
2. Yes

999. Don’t know

996. Refuse to answer

XD6 [If XD5=1 (using contraception)] what are you doing to avoid pregnancy? *(Select all that apply)*

1. Male condom
2. Pill
3. Injection
4. Female condom
5. Foam/jelly
6. Periodic abstinence/rhythm
7. Withdrawal
8. Emergency contraception
9. Male sterilization
10. IUD/Coil
11. Implant

997. Other (specify)

996. Refuse

XD6.oth. [If XD6=997 other] what other method did you use?

_________________________________________________________

XD7. [If XD5=0 (not using contraception)]Young people have different reasons for not using contraceptives (birth control). Tell me which reasons best describe why you and [response XB1] did not use birth control? *(Select as many as apply)*

XD7_1 I was too embarrassed to talk about using birth control

XD7_2 I wanted to get pregnant

XD7_4 Things were out of control

XD7_5 It was too hard to get [response XB1] to use birth control with me

XD7_6 Birth control interferes with enjoyment

XD7_7 I didn’t know where to get birth control

XD7_8 I didn't want to seem too eager for sex

XD7_9 I didn't think I could get pregnant

XD7_10 I never really thought about it

XD7_11 I can’t afford it

1. No
2. Yes

999. Don’t know

996. Refuse to answer

XD8. Do you think that you will have sexual intercourse with someone in the next year?

1. No
2. Yes
3. Maybe

999. Don’t know

996. Refuse to answer

XD9 Here are some statements about adolescent pregnancy. Please indicate whether you agree a lot, agree a little, disagree a little, or disagree a lot.

XD9_1 Adolescent girls who get pregnant should have an abortion if they are not married

XD9_2 Adolescent girls who get pregnant should have an abortion because they are too young to raise children

XD9_3 Adolescent girls who get pregnant should have an abortion to stay in school

1. Agree a lot
2. Agree a little
3. Neither agree, nor disagree
4. Disagree a little
5. Disagree a lot

996. Refuse to answer

**XI. Empowerment**

**X.A. Freedom of movement**

XIA1. Can you tell me how often you are allowed to do the following alone (without an adult present)?

XIA1a Go to after-school activities (like sports clubs)

XIA1b Go to a party with BOYS and GIRLS

XIA1c Meet with friends after school

XIA1d Go to community center/movies/youth center

XIA1e Go to church/mosque/temple or religious center

XIA1f Visit a friend of the opposite sex (i.e visit a boy)

1. Never
2. Rarely
3. Sometimes
4. Often

999. Don’t know

996. Refuse to answer

**XI.B. Voice**

XIB1. How often are the following statements true for you?

XIB1a My parents or guardians ask for my opinion on things

XIB1b My parents or guardians listen when I share my opinion

XIB1c My friends ask my advice when they have a problem

XIB1d If I see something wrong in school or the neighborhood I feel I can tell someone and they will listen

XIB1e I can speak up in class when I have a comment or question

XIB1f I can speak up when I see someone else being hurt

XIB1g I can ask adults for help when I need it

1. Never
2. Rarely
3. Sometimes
4. Often

999. Don’t know

996. Refuse to answer

**XI.C. Behavioral Control/Decision-Making**

XIC1. How often are you able to make each of the following decisions on your own, without an adult?

XIC1a What clothes to wear when you are not in school/working?

XIC1b What to do in your free time

XIC1c What to eat when you are not at home

XIC1d How much education you will get (e.g., go to secondary school, university)

XIC1e Who you can have as friends

XIC1f Decide WHEN to marry on your own

XIC1g Decide WHO you will marry on your own

1. Never
2. Rarely
3. Sometimes
4. Often

999. Don’t know

996. Refuse to answer

**XI.D Economic Empowerment**

XID1. Some young people take up jobs for which they are paid. Others sell things, have a small business or do work for family or neighbors for which they make some money. Over the past 6 months have you done any chores or activities for which you got paid money?

1. No
2. Yes

999. Don’t know

996. Refuse to answer

*(→ If* XID1=0 *(has not made money), skip to* XID4*)*

XID2. What did you do in the last 6 months to earn money? (Check as many as apply)

1. Day labor or temporary work
2. Worked for family (such as parents or relatives)
3. Worked for neighbors or friends
4. Doing occasional jobs for pay
5. Providing services (such as babysitting, hair styling, nail art, selling things like water or cigarettes, washing cars)
6. Begging, panhandling or garbage-picking
7. Part- or full-time job
8. Got an allowance from [response IIA1] or another caretaker

997. Other _______

996. Refuse to answer

XID3. Whose idea was it that you work to earn money? *(Select all that apply)*

1. My idea alone
2. Mother/father
3. Boyfriend/girlfriend
4. Other friend
5. Other family member

997. Other _______

996. Refuse to answer

X1D3oth. [If X1D3=997 other] Which other person suggested that you work to earn money?

_________________________________________________________

XID4. In the past 6 months have you received gifts of money? (Including from a boyfriend or girlfriend)?

1. No
2. Yes

999. Don’t know

996. Refuse to answer

*(→ If* XID4=0 *(has not received gifts of money), skip to* XII*)*

XID5. If you did get money over the past 6 months where did you get the money from? *(Select all*

*that apply)*

1. Mother/father
2. Boyfriend/girlfriend
3. Other friend
4. Other family member
5. Teacher/School

997. Other

996. Refuse to answer

X1D5oth. [If X1D6=997 other] Which other person gave you money.

_________________________________________________________

XID6. Who decided how the money you earned/received over the past 6 months was spent?

*(Select all that apply)*

1. Me
2. Mother/father
3. Boyfriend/girlfriend
4. Other friend
5. Other family member
6. There was never a discussion; it was expected that I give it to my family

997. Other

996. Refuse to answer

X1D6oth. [If X1D5=997 other] Which other person decided how the money you earned or received was spent.

_________________________________________________________

**XII. Future Expectations**

In this section we would like to better understand at what age you expect certain things will happen to you in your life.

XII1. I will read some other things that happen in the life of most people. Please tell me if you

think they will happen to you:

XII1a First period

XII1c Leave school forever

XII1d First child

XII1e First job

XII1f Marry

1. Will never happen
2. Will happen
3. Has already happened

999. Don’t know

996. Refuse to answer

XII2. If XII1n is “Will happen”: At what age do you think these things will happen to you?

XII2a First period

XII2c Leave school forever

XII2d First child

XII2e First job

XII2f Marry

1. 10-14
2. 15-19
3. 20-24
4. 25-29
5. 30 or more

999. Don’t know

996. Refuse to answer

XII31. [If XII1d IS NOT= 0] How many children do you think that you will have in your lifetime?

NUMERIC

1. None

999. Don’t know

996. Refuse to answer

*“We are now finished with the questions. Thank you so much for talking to me, you did a really great job!”*

[Enter finalization code “2017”] to close out the survey

**Distress screening to be asked at the end of survey (link to respondent ID)**

[CHECK IXB1m =1 or 2: Interviewer note: Respondent reported abuse

dstrss1. D1. “I know the some of the questions that I asked may have been sensitive or uncomfortable for you to talk about. Can you tell me how are you feeling right now?”

[Interviewer should fill out the below options based on what the respondent tells them (e.g. ok, good, worried, upset) or their impression of how the respondent is feeling]

1. Good (happy, not at all upset)
2. Ok (not happy or upset)
3. Somewhat worried/upset
4. Very worried/upset
5. Reported abuse

dstrss1a. [If dstrss1=3 or 4, ask:] If you are comfortable telling me, please tell me what has upset, worried or made you uncomfortable? (string)

_______________________________________________________________

dstrss1b. If abuse was reported [dstrss1=5], please specify/give more details

________________________________________________________________

dstrss2. [If dstrss1=4 or 5, ask:] D2. If the respondent reports abuse or that they are very worried/upset:

“Based on your saying to me [or showing] that our interview may have upset you, I would like to share this with my supervisor [field coordinator] so that we can let you know where to find help that might be useful. [For adolescents] If ok with you, we will also talk to your mother (or father) so that they can help you” [If the adolescent does not want to share this with his/her parents/guardians, help them identify another adult they could talk with].

Action taken: __________________________________________________________

dstrss3. D3. If the respondent does not report abuse and is not very worried/upset [dstrss1=1 or 2]:

“We have talked about many things today that you might have more questions about [give examples e.g. romantic relationships, sex, bullying or violence]. I want to give you this card with numbers and locations for organizations [say the local names] that work with young people your age. You might have heard of some, and some might be new to you. If you have questions or want to talk to someone, you can call them and they will try to help you.”

**Interviewer assessment**

Interviewer, please complete the questions below based on your own observation and assessment of the entire interview process, and the respondent.

dstrss4. D4. How did you find the respondent’s cooperation?

1. Very good
2. Moderate (ok)
3. Bad
4. Very bad

dstrss4a. [If dstrss4=4] Please explain why very bad (string)

______________________________________________

dstrss5. D5. How accurate/true did you find the respondent’s answers?

1. Very accurate/true
2. Somewhat accurate/true
3. Not very accurate/true
4. Highly inaccurate (the responses should not be trusted)

dstrss5a [If dstrss5=4] Please explain why can’t be trusted (string)

_________________________________________________

dstrss6. D6. How did you find the respondent’s understanding of the questions discussed?

1. Very good (understood perfectly)
2. Moderate (understood ok)
3. Bad (did not understand many of the questions)
4. Very bad (did not understand at all)

dstrss6a. [If dstrss6=4] Please explain about their not understanding (string)

_____________________________________________

dstrss7. D7. How did you find the respondent’s concentration and attentiveness during the

interview?

1. Very good (highly concentrated/attentive)
2. Moderate/ok (somewhat concentrated/attentive)
3. Bad (could not concentrate for many parts of the interview)
4. Very bad (could not concentrate at all)

dstrss7a. [If dstrss7=4] Please explain why very bad at concentration (string)

___________________________________________________

dstrss8. D8. About how many breaks did you take during the full interview?

______ Number of breaks

dstrss9. D9. [If A9=2 – 11 (interview not completed)] Other comments about the interview ____________________________________________________

This concludes our interview. Thank you very much for your time
