# Supplementary material for: Reaching early adolescents with a complex intervention for HIV prevention: findings from a cohort study to evaluate DREAMS in two informal settlements in Nairobi, Kenya
Source: BMC Public Health. 2021 Jun 10;21:1107. doi: 10.1186/s12889-021-11017-y (PMC8194171; doi:10.1186/s12889-021-11017-y)
Supplement: Supplementary file 4 — Additional file 4. Uptake* of each intervention by round of interview. *Participation in the intervention in the last 12 months prior to date of interview; N*-The denominator for 2018 is those seen in 2018, as some individuals seen in 2019 were not seen in 2018. [file 12889_2021_11017_MOESM4_ESM.pptx]

## Slide 1
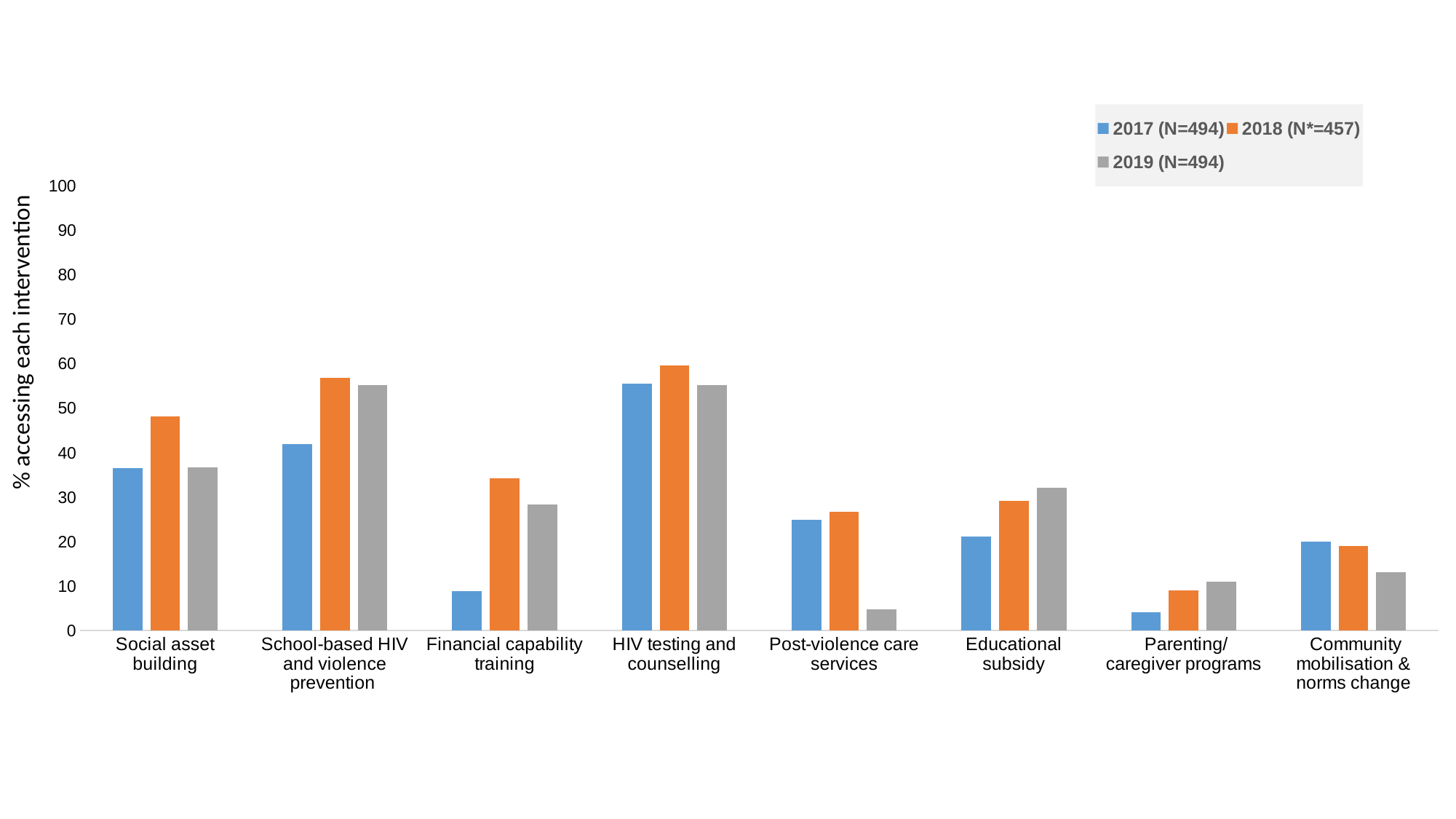

### Chart
| Category | 2017 (N=494) | 2018 (N*=457) | 2019 (N=494) |
|---|---|---|---|
| Social asset building | 36.4 | 48.1 | 36.6 |
| School-based HIV and violence prevention | 41.9 | 56.7 | 55.1 |
| Financial capability training | 8.9 | 34.1 | 28.3 |
| HIV testing and counselling | 55.5 | 59.5 | 55.1 |
| Post-violence care services | 24.9 | 26.7 | 4.7 |
| Educational subsidy | 21.1 | 29.1 | 32.0 |
| Parenting/caregiver programs | 4.0 | 9.0 | 11.0 |
| Community mobilisation & norms change | 20.0 | 19.0 | 13.0 |% accessing each intervention
