# Supplementary material for: Reaching early adolescents with a complex intervention for HIV prevention: findings from a cohort study to evaluate DREAMS in two informal settlements in Nairobi, Kenya
Source: BMC Public Health. 2021 Jun 10;21:1107. doi: 10.1186/s12889-021-11017-y (PMC8194171; doi:10.1186/s12889-021-11017-y)
Supplement: Supplementary file 5 — Additional file 5. Layering of primary interventions: number of EAG who accessed each primary intervention and its combinations cumulatively by 2019 (N* = 456). *Number who accessed at least one primary intervention by 2019. [file 12889_2021_11017_MOESM5_ESM.pptx]

## Slide 1
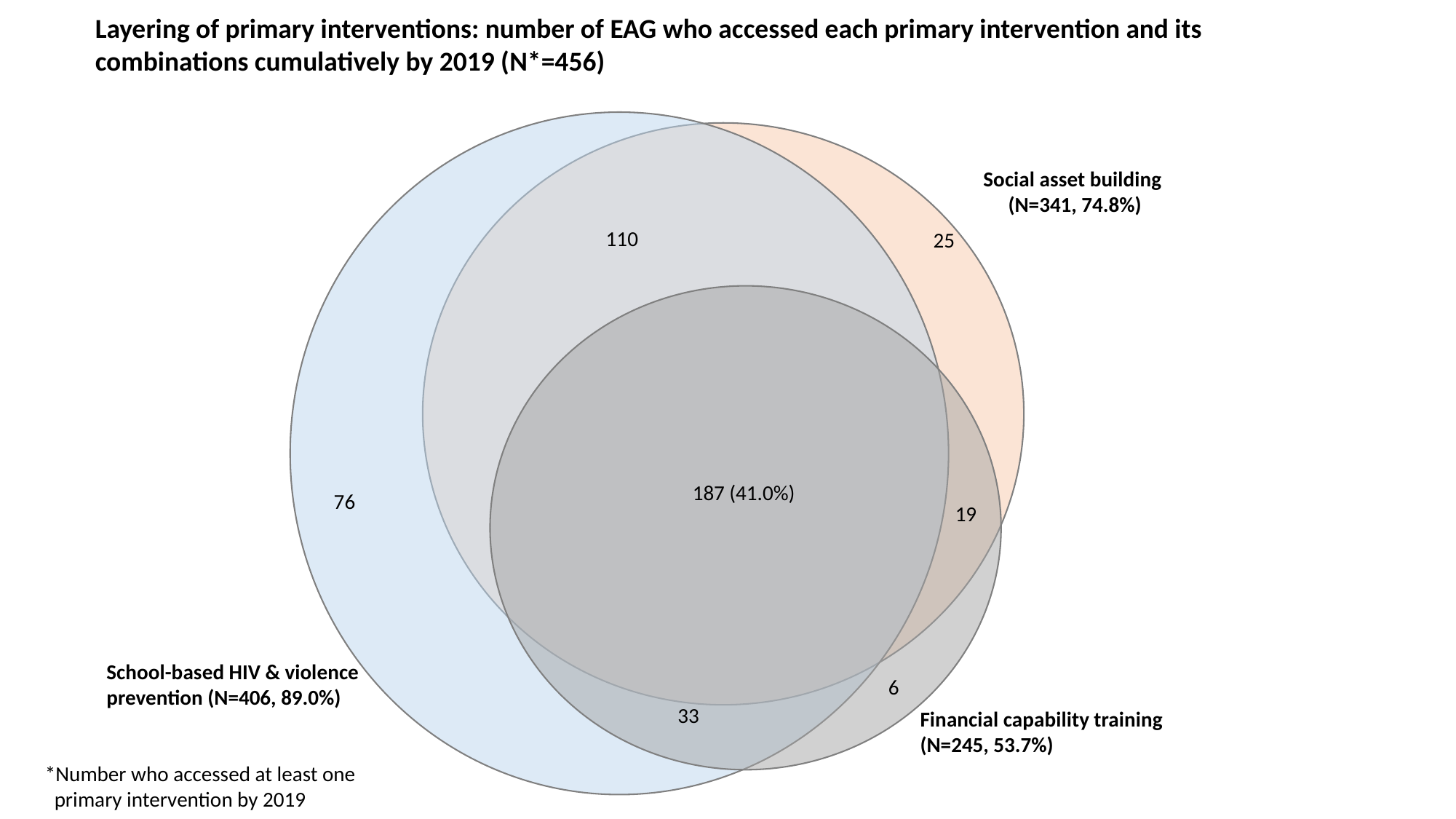

Layering of primary interventions: number of EAG who accessed each primary intervention and its combinations cumulatively by 2019 (N*=456)
Social asset building
(N=341, 74.8%)
110
25
187 (41.0%)
76
19
School-based HIV & violence prevention (N=406, 89.0%)
6
33
Financial capability training (N=245, 53.7%)
*Number who accessed at least one
 primary intervention by 2019
