# Supplementary material for: Reaching early adolescents with a complex intervention for HIV prevention: findings from a cohort study to evaluate DREAMS in two informal settlements in Nairobi, Kenya
Source: BMC Public Health. 2021 Jun 10;21:1107. doi: 10.1186/s12889-021-11017-y (PMC8194171; doi:10.1186/s12889-021-11017-y)
Supplement: Supplementary file 7 — Additional file 7. Layering of individual and contextual level interventions: number of EAG who accessed at least one intervention across individual and contextual levels cumulatively by 2019 (N* = 485). *Number who accessed at least one intervention from any level by 2019. [file 12889_2021_11017_MOESM7_ESM.pptx]

## Slide 1
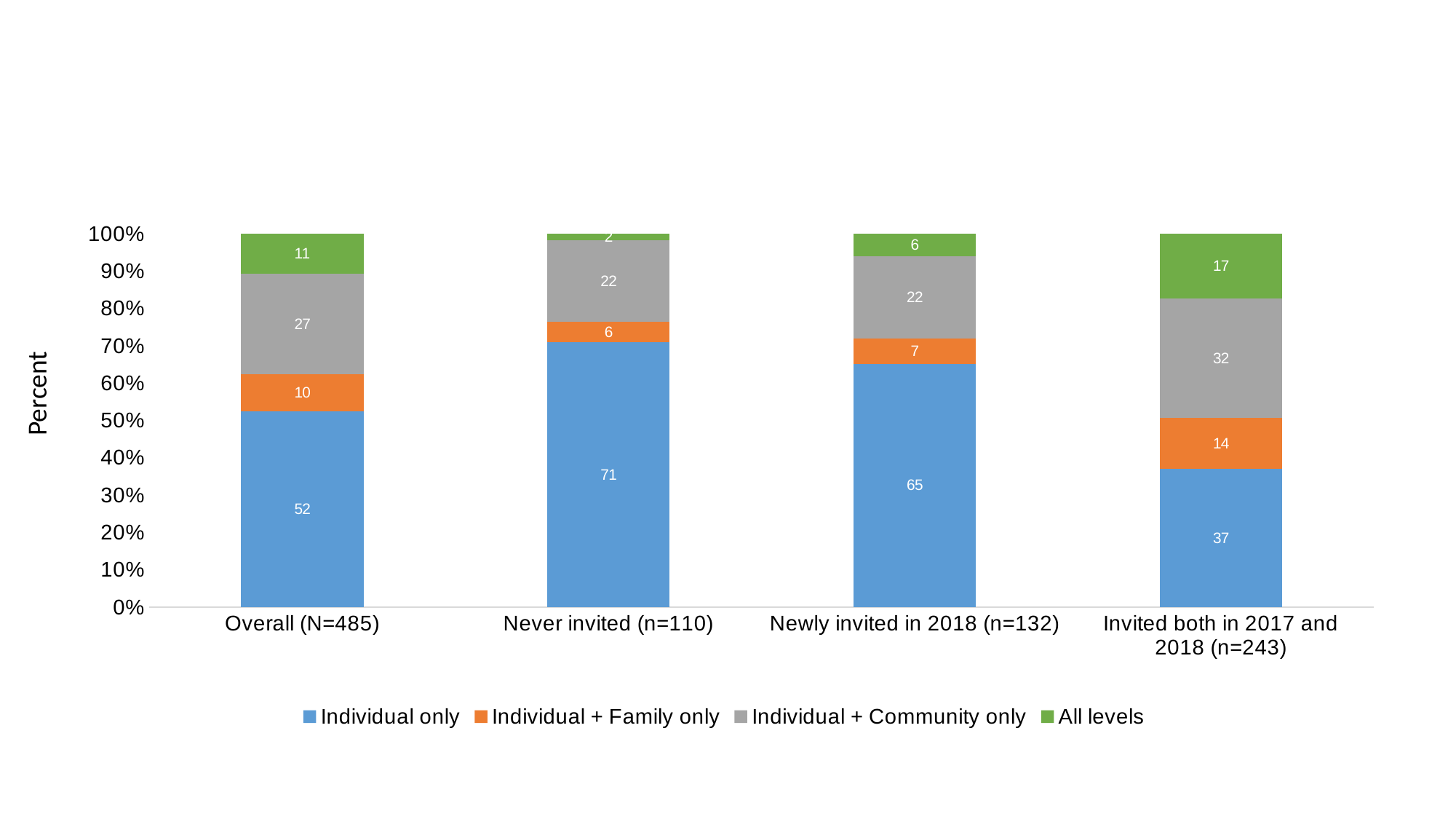

### Chart
| Category | Individual only | Individual + Family only | Individual + Community only | All levels |
|---|---|---|---|---|
| Overall (N=485) | 52.4 | 9.9 | 27.0 | 10.7 |
| Never invited (n=110) | 70.9 | 5.5 | 21.8 | 1.8 |
| Newly invited in 2018 (n=132) | 65.2 | 6.8 | 22.0 | 6.1 |
| Invited both in 2017 and 2018 (n=243) | 37.0 | 13.6 | 32.1 | 17.3 |Percent
